# Analysis of Putative Apoplastic Effectors from the Nematode, *Globodera rostochiensis*, and Identification of an Expansin-Like Protein That Can Induce and Suppress Host Defenses

**DOI:** 10.1371/journal.pone.0115042

**Published:** 2015-01-21

**Authors:** Shawkat Ali, Maxime Magne, Shiyan Chen, Olivier Côté, Barbara Gerič Stare, Natasa Obradovic, Lubna Jamshaid, Xiaohong Wang, Guy Bélair, Peter Moffett

**Affiliations:** 1 Département de Biologie, Université de Sherbrooke, 2500 Boulevard de l’Université Sherbrooke, Sherbrooke, Québec, Canada; 2 Horticulture R & D Centre Agriculture and Agri-Food Canada, 430 Boulevard Gouin, St-Jean-sur-Richelieu, Québec, Canada; 3 Department of Plant Pathology and Plant-Microbe Biology, Cornell University, Ithaca, New York 14853, United States of America; 4 Agricultural Institute of Slovenia, Plant Protection Department, Hacquetova ulica 17, SI-1000 Ljubljana, Slovenia; 5 Robert W. Holley Center for Agriculture and Health, US Department of Agriculture, Agricultural Research Service, Ithaca, New York 14853, United States of America; Leibniz-Institute for Vegetable and Ornamental Crops, GERMANY

## Abstract

The potato cyst nematode, *Globodera rostochiensis*, is an important pest of potato. Like other pathogens, plant parasitic nematodes are presumed to employ effector proteins, secreted into the apoplast as well as the host cytoplasm, to alter plant cellular functions and successfully infect their hosts. We have generated a library of ORFs encoding putative *G. rostochiensis* putative apoplastic effectors in vectors for expression *in planta*. These clones were assessed for morphological and developmental effects on plants as well as their ability to induce or suppress plant defenses. Several CLAVATA3/ESR-like proteins induced developmental phenotypes, whereas predicted cell wall-modifying proteins induced necrosis and chlorosis, consistent with roles in cell fate alteration and tissue invasion, respectively. When directed to the apoplast with a signal peptide, two effectors, an ubiquitin extension protein (*Gr*UBCEP12) and an expansin-like protein (*Gr*EXPB2), suppressed defense responses including NB-LRR signaling induced in the cytoplasm. *Gr*EXPB2 also elicited defense response in species- and sequence-specific manner. Our results are consistent with the scenario whereby potato cyst nematodes secrete effectors that modulate host cell fate and metabolism as well as modifying host cell walls. Furthermore, we show a novel role for an apoplastic expansin-like protein in suppressing intra-cellular defense responses.

## Introduction

Plants are hosts for microbial and viral pathogens, as well as for multicellular parasites such as insects, parasitic plants and nematodes. Despite the many biological differences between microbial and multicellular pathogens, many of the principles governing the molecular interactions between these pathogens and their hosts are thought to be similar. Plants detect microbes through the recognition of pathogen-associated molecular patterns (PAMPs), by membrane spanning proteins known as pattern-recognition receptors (PRRs). These PRRs induce PAMP-triggered immunity (PTI) by triggering signaling cascades that initiate mitogen-activated protein kinase (MAPK) cascades, production of reactive oxygen species (ROS), production of antimicrobial compounds, expression of pathogenesis related *(PR)* genes and localized deposition of callose [1].

To overcome PTI, host-adapted pathogens employ secreted proteins known as effectors to promote infection. Many effectors are delivered to the host cytoplasm and a number of these have been shown to interfere with intracellular signaling pathways induced by PTI mechanisms [2–4]. Many pathogens also deliver effector proteins to the plant apoplast, some of which have also been show to promote pathogenesis, either by masking PAMP recognition or by directly inhibiting host apoplastic defense proteins [5–10].

Effector proteins can also induce effector-triggered immunity (ETI) by virtue of their being recognized by the nucleotide-binding and leucine-rich repeat (NB-LRR) proteins encoded by disease resistance (R) genes [11]. NB-LRR proteins recognize effectors delivered to the host cytoplasm and induce a much stronger response than PTI, often associated with a type of cell death known as the hypersensitive response (HR). Apoplastic effectors can also induce ETI by being recognized by receptor-like proteins (RLPs) present in the plant plasma membrane [12,13].

The potato cyst nematode (PCN), *Globodera rostochiensis*, is an obligate biotroph and parasitizes crops such as potato, tomato and eggplant. After hatching from eggs in the soil, cyst nematodes penetrate the roots as infective juveniles and move intracellularly into the root cortex. Phytopathogenic nematodes secrete effector proteins through a hollow stylet either into the cytoplasm of the host cell or into the apoplast [14]. Like microbial effectors, several nematode effectors have been shown to inhibit plant defense responses and to be recognized by NB-LRR and RLP proteins [13,15,16]. Unlike most plant pathogens however, nematodes also induce dramatic changes in cell identity and metabolism. Cyst nematodes induce the development of a specialized feeding structure called a syncytium, a large, multinucleate, and metabolically active cell that provides nutrients to the developing nematode. The syncytium expands as a result of directed local cell wall break down of the initial syncytial and neighboring cells and subsequent fusion of the protoplast [17]. These alterations are presumed to be mediated, in large part, by secreted nematode effector proteins [14].

Most secreted effector-like proteins produced by cyst nematodes are believed to be produced in the pharyngeal gland cells (two subventral and one dorsal), and are thought to be delivered to the host through the stylet [18]. Subventral glands are highly active during the penetration and migratory stage of parasitism and tend to produce effector proteins presumed to function in the apoplast, such as cell wall-modifying proteins [14]. In the sedentary stage, the subventral glands shrink in size while the dorsal gland enlarges and becomes active during syncytium formation and maintenance [19]. Effectors produced in the latter gland are thought to be delivered to the host cytoplasm [14].

Several approaches have been used to identify effector proteins from nematodes, including cDNA-AFLP, microarrays, EST mining, proteomics and candidate gene approaches [14]. With other pathogens, local and systemic expression of effectors *in planta* via viral vectors has been shown to be an effective method to identify effectors that cause dramatic phenotypes in plants that may be indicative of their importance in pathogenesis. For example, the crinkler (CRN) family of proteins in *Phytophthora infestans* were defined by such a strategy and have since been shown to form a major class of effectors in all oomycetes [20].

We have identified at least fourteen putative *G. rostochiensis* apoplastic effector proteins from public databases. When transiently expressed by agroexpression and/or a potato virus X (PVX) expression vector in different solanaceous plants, approximately half of the effectors caused phenotypes, including the induction of cell death, chlorosis and dwarfing as well as developmental phenotypes. In addition, the ubiquitin extension protein *Gr*UBCEP12, as well as an expansin-like protein *Gr*EXPB2, also showed the potential to suppress defense-related cell death in *Nicotiana benthamiana* and/or *N. tabacum*. These extracellular effectors were able to inhibit the resistance mediated by the signaling induced by the N and Rx NB-LRR proteins in *N. benthamiana*. The *Gr*EXPB2 protein also elicited defense responses in different plant species. Our results demonstrate that apoplastic effector proteins can affect intra-cellular signaling pathways and suggest novel functions for expansin-like proteins in plant-nematode interactions.

## Material and Methods

### Bacterial strains, plants growth conditions

Expression vectors based on PVX were delivered using *Agrobacterium tumefaciens* strain GV3101 by infiltration or toothpick inoculation [21]. All other binary vectors were delivered by Agro-infiltration using strain C58C1 as previously described [22]. All plants were grown at 22°C, 50% humidity in controlled growth chamber condition with 14h/10h light/dark cycle.

### Construction of Gateway compatible plasmids

The gateway cassette with terminal *attR* recombination sites and *ccdB* selection gene was amplified by PCR from the vector pGBKCg with a forward primer including restriction sites *XbaI* and *ClaI* and a reverse primer containing an *Xho1* site (S3 Table). The resulting PCR product was ligated into the *SmaI* site of pEAQ_35SE (Brosseau and Moffett unpublished) a derivative of pEAQSelectK [23] to make pEAQ_35SE-Gw (pEAQ35S). The gateway cassette was subsequently excised from pEAQ35S with *XhoI* and *ClaI* and cloned into the *ClaI* and *SalI* sites of pGR106 and pGR103 [21] to make pGR106-Gw (PVX) and pGR103-Gw (PVX-HB) respectively.

### Identification and amplification of candidate secreted effector protein

Candidate secreted effector proteins (CSEPs) were identified by searching all *Globodera rostochiensis* predicted ORFs on NCBI and EST databases (http://www.nematodes.org/downloads/databases/NEMBASE4/GRC_nuc.fsa). The resulting collection of ORFs was investigated for the presence of N-terminal signal peptides in the predicted proteins using SignalP version 3.0 [24]. All proteins with a predicted signal peptide (SP) identified by hidden markov models (HMM) algorithm of SignalP version 3.0 http://www.cbs.dtu.dk/services/SignalP/) [24] were retained as potential candidate effectors. Proteins not predicted to be secreted by SignalP but referenced as secreted in the literature were also kept as CSEPs. To verify further, a pipeline of bioinformatics tools and software; TargetP, TMHMM and ProtComp as described previously [25] were used for the prediction of CSEPs.

### Cloning of effectors and expression *in planta*

Pre-parasitic second-stage juveniles (pre-J2s) of *G. rostochiensis* or infected potato roots containing different nematode parasitic stages from Québec populations [26] were used for RNA isolation using Trizol as previously described [27]. mRNA was converted to cDNA by RT-PCR using an oligo dT primer and the superscript CellsDirect cDNA synthesis system (Invitrogen life technology). Selected genes were amplified with specific primers (S3 Table) using high fidelity KOD hot start DNA polymerase (EMD Millipore). Sense primers were designed to amplify the effectors including, at their 5′ end, the *attB1* sequence (GGGGACAAGTTTGTACAAAAAAGCAGGCTTC) followed by a Kozak consensus sequence and a start codon (AGAACCATG). Reverse primers contained the *attB2* sequence (GGGGACCACTTTGTACAAGAAAGCTGGGTC) followed by the gene-specific sequence including the native stop codon. Effector sequences differing from previously published sequences are listed in S2 Table and have been deposited in Genbank (accessions KF963513-KF963529) and are shown in S5 Fig.

PCR products were cloned into pDONR207 or pDONR221 using BP clonase and recombined into the gateway compatible binary vector pEAQ35S, PVX and PVX-HB by LR clonase reaction (Invitrogen) following the manufacturer’s instructions.

Four to six week-old *N. tabacum, N. benthamiana*, potato and tomato were used for agroinfiltration and agroinfection as previously described [21,28].

### Cell death and disease resistance suppression assays

For cell death suppression assays, *Agrobacterium* strains carrying the CSEP either in the pEAQ35S or PVX constructs were diluted in 10 mM MgCl_2_ such that all effectors were infiltrated at a final OD_600_ of 0.2 and the cell death inducers and P38, the viral suppressor of RNA silencing of Turnip Crinkle Virus (TCV) [29], at a final OD_600_ of 0.1. A control with the cell death inducer and empty vector was always infiltrated on the opposite side of the leaf. All experiments were repeated at least three times. Cell death symptoms were scored 3–5 DPI and pictures were taken 5 DPI. Cell death suppression was assessed visually on a scale of 0 to 2. A complete absence of cell death was given a score of 2 while partial suppression was given a score of 1 and no suppression was attributed a score of zero. The overall suppression activity for suppressors was calculated from 12–15 infiltration sites. The scores from all infiltrated sites for a particular effector were added together and divided by the theoretical maximum score for that effector (i.e. the number of assays times two) to obtain a percentage cell death score.

PVX resistance assays induced by Rx or by N were performed as previously described [30,31].

### Gene expression analysis by quantitative real-time RT-PCR (qRT-PCR) assays

RNA extractions and qRT-PCR assays (iQ SYBR Green Supermix; Bio-Rad Laboratories) were performed using primers listed in S3 Table as previously described [32,33]. mRNA samples for each developmental stage were prepared from two independent experiments and used for cDNA synthesis. All qPCR assays consisted of three technical replicates for each cDNA sample. The *G. rostochiensis β-actin* gene (*Gract-1*) (EF437156) was used as an endogenous reference for data analysis using the 2^−ΔΔCt^ method [34]. For each developmental stage, 2^−ΔΔCt^ represented the amount of the target gene expression that was normalized to *Gract-1* and relative to a calibrator that had the lowest expression in the cyst or other life stage.

## Results

### Identification of candidate secreted effectors proteins (CSEPs) in *G. rostochiensis*

A common property of eukaryotic pathogen effectors is that they are secreted proteins, containing an N-terminal signal peptide (SP), regardless of whether their eventual location of action is in the cytoplasm or the apoplast [3]. From more than 373 *G. rostochiensis* sequences obtained from NCBI and the nematode EST database, NEMBASE4, we identified thirty-seven proteins having a putative SP. Of the proteins identified, many were known to be probable intracellular effectors (e.g. SPRYSEC proteins) and these will be described elsewhere. Several candidate ORFs are predicted to encode enzymes with pectate lyase, endoglucanase, glutathione peroxidase and metalloproteinase activities based on homology and previous reports [35,36]. Due to their predicted functions, these proteins are very likely to function in the apoplast (Table 1). In agreement with this, homologues of many of these proteins (S1 Table), have been reported either as being expressed in the esophageal glands and/or being secreted into the apoplast [13, 37,38]. Apoplastic effectors are often cysteine rich and we found that ten out of twenty-three of these proteins have four or more Cys residues in the mature protein (S1 Table). We also included in this study a number of effectors of unknown function whose location of action could not be predicted (Table 1).

**Table 1:** Description of cloned apoplastic secreted effector proteins.

**Effector**	**Predicted protein**	**Predicted localization**	**Predicted or known function**	**Phenotype observed *in planta***
*Gr*CLE1	CLE-like peptide	Apoplastic	Mimicking plant CLE peptides, involved in syncytium formation	downward curving leaves and chlorosis in *N. benthamiana* ^¶^
*Gr*CLE-4A	CLE-like peptide	Apoplastic	Mimicking plant CLE peptides, involved in syncytium formation	No visible phenotype
*Gr*CLE-4D	CLE-like peptide	Apoplastic	Mimicking plant CLE peptides, involved in syncytium formation	downward curving leaves and chlorosis in *N. benthamiana* ^¶^
*Gr*CLE-4B	CLE-like peptide	Apoplastic	Mimicking plant CLEs peptides, involved in syncytium formation	downward curving leaves and chlorosis in *N. benthamiana* ^¶^
*Gr*CLE-4C	CLE-like peptide	Apoplastic	Mimicking plant CLE peptides, involved in syncytium formation	downward curving leaves and chlorosis in *N. benthamiana* ^¶^
*Gr*EXPB1	Expansin-like protein	Apoplastic	Cell wall extension	No visible phenotype
*Gr*EXPB2	Expansin-like protein	Apoplastic	Cell wall extension	Chlorosis and dwarfing in *N. benthamiana* ^¶^; necrosis in tomato and potato; and suppression of host defense
*Gr*VAP1	Venom allergen protein	Apoplastic	Defense, potential avirulence protein	No visible phenotype
*Gr*ENG1	Endoglucanase	Apoplastic	Cell wall modification	No visible phenotype
*Gr*ENG2	Endoglucanase	Apoplastic	Cell wall modification	No visible phenotype
*Gr*ENG3	Endoglucanase	Apoplastic	Cell wall modification	No visible phenotype
*Gr*PEL1	Pectate lyase	Apoplastic	Plant cell wall degradation	Severe malformation in the leaves and death at the end in *N. benthamiana* ^¶^; dwarfing and necrosis in tomato
*Gr*PEL2	Pectate lyase	Apoplastic	Plant cell wall degradation	Severe malformation in the leaves and death in *N. benthamiana* ^¶^; dwarfing and necrosis in tomato
*Gr*MTP	Metalloprotease	Apoplastic	Protein degradation	Curling leaves and necrotic collapse of the leaves^¶^
*Gr*GPX	detoxification of ROS	Unknown	Detoxification of ROS and plant defense suppression	No visible phenotype
*Gr*TPX	detoxification of ROS	Unknown	Detoxification of ROS and plant defense suppression	No visible phenotype
*Gr*AMS1	Sensory protein	Unknown	Sensory protein, help in location of the host	No visible phenotype
*Gr* SXP1	Unknown	Unknown	Unknown	No visible phenotype
*Gr*4D06	Unknown	Unknown	Unknown	No visible phenotype
*Gr*E9	Unknown	Unknown	Unknown	No visible phenotype
*Gr*A42	Unknown	Unknown	Unknown	No visible phenotype
*Gr*UBCEP12	Ubiquitin extension-like peptide	Nuclear and cytoplasmic, apoplastic	Feeding cell formation and plant defense suppression	shrunken downward curling leaves and succumbing to a necrotic collapse^¶^ and Suppression of host defense* ^§^
*Gr*SKP-1	Ubiquitin ligase component	Cytoplasmic, apoplastic	Involved in signal transduction, protein degradation	Bushy plant with smaller leaves in *N. benthamiana* ^¶^

*Transient expression in *N. benthamiana*

^§^Transient expression in *N. tabacum*

^¶^Systemic expression in *N. benthamiana* via PVX

We conducted WUBLASTP searches against the genome sequence of *G. pallida* [39], a close relative of *G. rostochiensis*, and the genus *Heterodera* that includes cyst nematodes of soybean, sugar beet and cereals. As expected, all of the candidate effectors have a homolog in *G. pallida* with protein identities of 37–97% (S1 Table). Several effectors also have a homolog in the genus *Heterodera* with protein identities of 31–85% (S1 Table). We also conducted BLASTX in GenBank excluding *Globodera* and *Heterodera* species. Eleven of the CSEPs also showed homology (with protein identity of ≥ 40%) to predicted effectors from plant parasitic nematodes outside the *Globodera* and *Heterodera* genera (S1 Table).

One of the identified CSEP-encoding genes, *GrExp1* appears to be the result of alternative splicing of *ExpB1*. Compared to *Gr*EXPB1, *Gr*EXP1 is missing a continuous stretch of ten amino acids in the C-terminal region and we were unable to amplify this isoform from cDNA of preparasitic second-stage juveniles (pre-J2s). Three other candidate effectors encoding genes, *GrCLE-4B1, GrCLE-4A3* and *GrENG-4* (S1 Table), appear to be alleles or copies of previously described genes *GrCLE4B, GrCLE4D* and *GrENG3*, respectively as they show only point mutation differences with published sequences.

Candidate ORFs were amplified by RT-PCR (S2 Table), followed by cloning and sequencing (see Materials and Methods for details). In our library of twenty-three CSEP clones, fourteen showed sequence differences with sequences reported in NCBI (S2 Table, S5 Fig.). Nine of these genes showed nonsynonymous amino acid variation, while four genes have synonymous mutations.

### Transcriptional profiles of several *G. rostochiensis* putative apoplastic CSEP-encoding genes

We used quantitative real-time RT-PCR (qRT-PCR) to determine the expression profile of four of the CSEP-encoding genes through the five nematode developmental stages: egg, pre-J2 and parasitic second-, third- and fourth-stage juveniles (par-J2, J3 and J4) (S1 Fig.). *GrPEL1* and *GrENG1* showed similar expression patterns, having consistent expression from egg to early parasitic stages. Expression of *GrExpB2* was highly upregulated in the pre-J2 stage, but significantly decreased in parasitic stages. In contrast to *GrExpB2* expression, *GrSkp1* showed dramatic and continuous upregulation in both early and late nematode parasitic stages.

### Construction of a *G. rostochiensis* CSEP library

**Figure 1:**
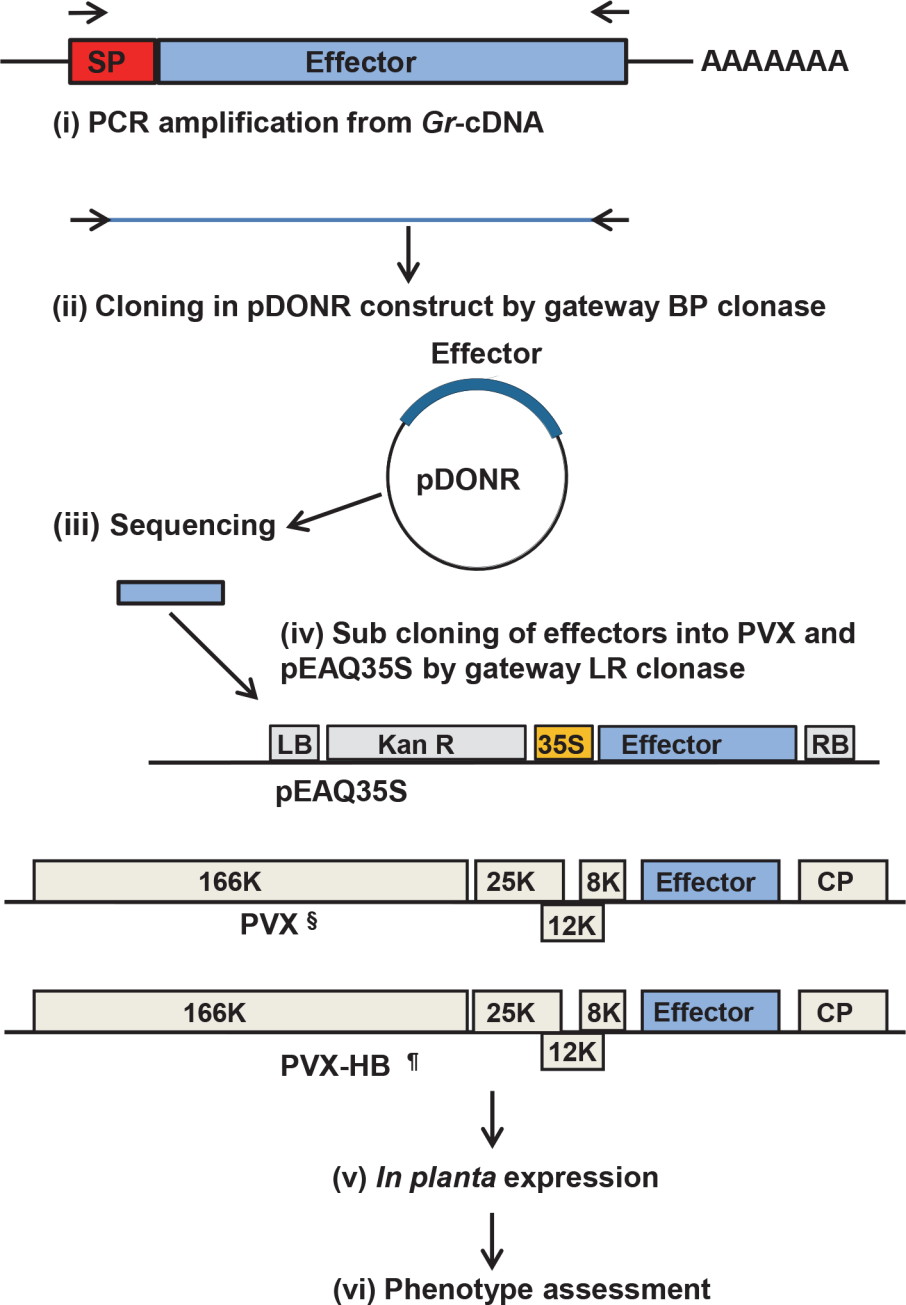
Cloning and expression strategy for putative *G. rostochiensis* effector proteins. Cloning and functional analysis of candidate secreted effector proteins *in planta* included; (i) PCR amplification of effector encoding genes from *G. rostochiensis* cDNA with their cognate SP (red); (ii) Cloning into the donor vector pDONR207 or pDONR221; (iii) Sequencing of multiple clones for sequence confirmation; (iv) Transfer of cDNAs from donor vector to gateway compatible pEAQ35S, PVX and PVX-HB, where ^§^ is Gateway compatible pGR106 and ^¶^ is Gateway compatible pGR103; (v) *In planta* expression of effectors by agroinfiltration and agroinfection; (vi) Identification of phenotype induced in different solanaceous plants and assessment for cell death suppression.

*G. rostochiensis* is an obligate biotroph for which no transformation system is available and loss- or gain-of-function mutant analysis is not possible in this organism at a high throughput level. To gain insight into the biological function of the *Gr*CSEPs we generated effector clone libraries in constructs for transient and systemic expression *in planta* (S2 Table). Effector ORFs were cloned in three different constructs: pEAQ35S; pGR106-Gw (herein referred to as PVX), and pGR103-Gw (expressing the Rx-breaking coat protein from PVX strain HB; herein referred to as PVX-HB) (Fig. 1). The latter was used for the expression of effectors in potato cultivars expressing the *Rx* gene [40]. CSEPs were transiently and systemically expressed in *N. benthamiana, N. tabacum*, tomato and potato both by agroinfiltration and agroinfection from binary vector pEAQ35S as well as from PVX vectors (Fig. 1). Phenotypes were assessed either for the induction of visual changes in plant health, morphology or architecture in systemically infected plants or in the induction of visible changes in agroinfiltrated leaf patches (Table 1).

### *Gr*EXPB2 induces chlorosis in *N. benthamiana* and cell death in tomato and potato

**Figure 2:**
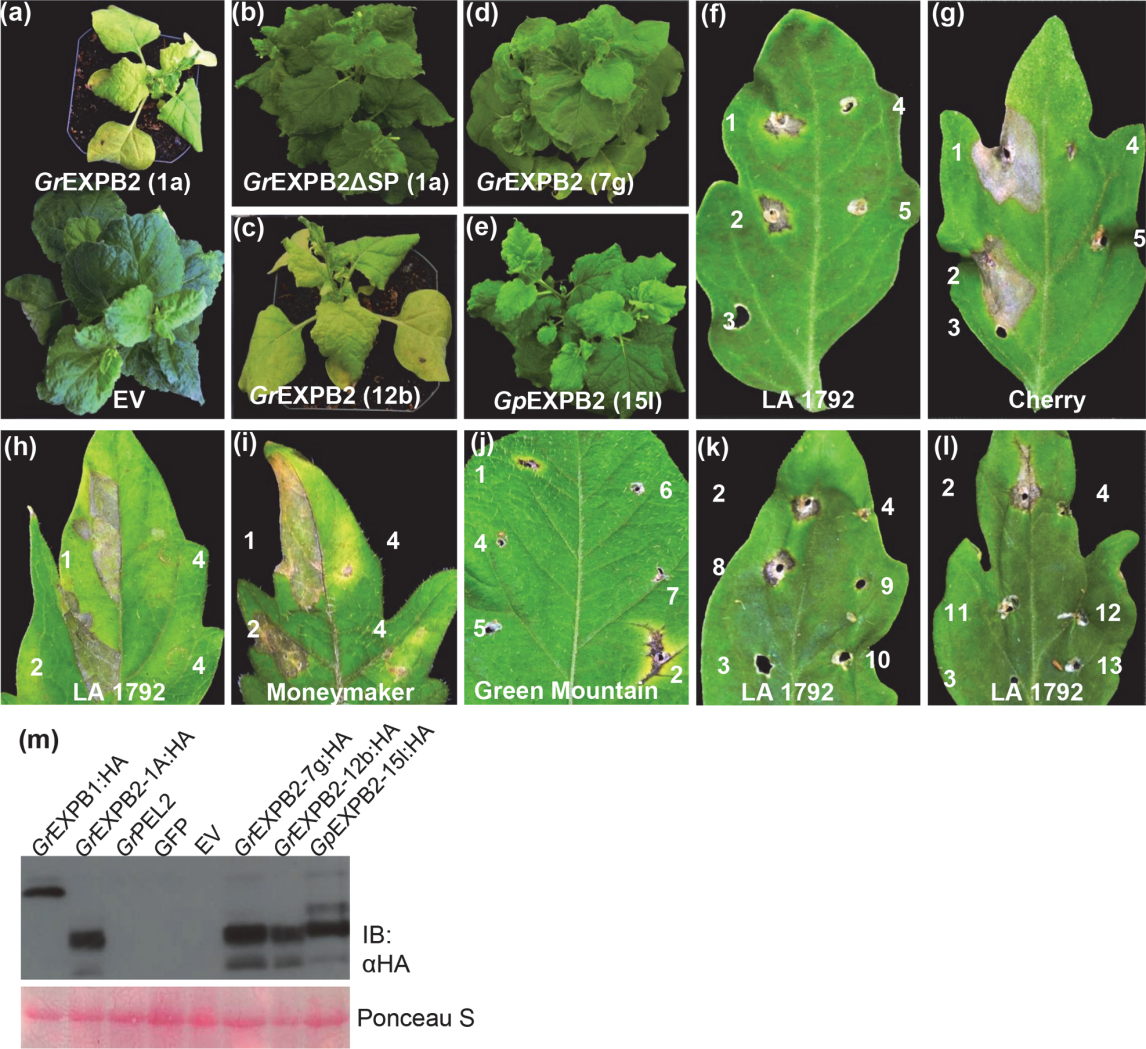
*Gr*EXPB2 induces necrosis in tomato and potato and chlorosis and dwarfing in *N. benthamiana*. *N. benthamiana* infected with (a) PVX-*Gr*EXPB2 clone 1A (top) or empty PVX vector (EV) (bottom). (b) PVX-*Gr*EXPB2ΔSP clone 1A, (c) PVX-*Gr*EXPB2:HIS-HA clone 12b, (d) PVX-*Gr*EXPB2:HIS-HA clone 7g, (e) PVX-*Gp*EXPB2:HIS-HA clone 15l. Plants were photographed at 20 DPI. Tomato cultivars (f, k, l) LA 1792 and (g) Cherry, were tooth pick inoculated with PVX derivatives or tooth pick only. The numbers correspond to 1, *Gr*EXPB2 clone 1A; 2, *Gr*EXPB2:HIS-HA clone 1A; 3, tooth pick only; 4, *Gr*EXPB2ΔSP clone 1A; 5, EXPB1:HIS-HA; 6, GFP; 7, *Gr*PEL1; 8, *Gr*EXPB2:HIS-HA clone 12b; 9, *Gp*EXPB2:HIS-HA clone 15l; 10, *Gr*EXPB2:HIS-HA clone 22j; 11, *Gr*EXPB2:HIS-HA clone 5a; 12, *Gr*EXPB2:HIS-HA clone 6b; and 13, *Gr*EXPB2:HIS-HA clone 7g. Leaves were photographed at 10 DPI. Tomato cultivars (h) LA 1792 and (i) Moneymaker were agroinfiltrated with pEAQ35S constructs expressing 1, *Gr*EXPB2 clone 1A; 2, AtRx and 4, Empty vector. Leaves were photographed at 6 DPI. Potato cultivar (j) Green Mountain was tooth pick inoculated with PVX constructs as in (f, g, k, l). Leaves were photographed at 15 DPI. (m) HA-tagged versions of EXPB2 variants, as indicated, were expressed in *N. benthamiana* leaves from the PVX vector and total protein extracts were prepared from infiltrated patches 4 DPI, followed by Anti HA immune blotting. Ponceau S staining (lower panel) was used to show equal loading.

Expansins are cell wall-loosening proteins that are involved in growth as well as in cell wall disassembly [41]. In nematodes, expansin-like proteins are thought to be secreted into the apoplast during invasion of the roots [42,43]. We identified two effectors, *Gr*EXPB1 and *Gr*EXPB2, predicted to encode expansin-like proteins, although *Gr*EXPB2 lacks a carbohydrate binding domain (CBD II) commonly found in expansin-like proteins (S2 Fig.). *Gr*EXPB2 induced dramatic symptoms when expressed systemically via PVX in *N. benthamiana*, including severe chlorosis and dwarfing (Fig. 2A). Consistent with its predicted apoplastic localization, *Gr*EXPB2-induced symptoms were completely abrogated when the SP was deleted (PVX-*Gr*EXPB2ΔSP; Fig. 2B), although we cannot rule out the possibility that deletion of this N-terminal region might affect protein stability or function. *Gr*EXPB2 has been reported to present a significant variability between individuals and between populations, although the most common sequence variant is the *Gr*EXPB2 “type” sequence reported here [44]. We expressed HA epitope-tagged versions of *Gr*EXPB2 “type” (12b), a variant *Gr*EXPB2 (7b) from an Ro5 population, an EXPB2 homolog from *G. pallida* (15l) [44] (S3 Fig.), as well as *Gr*EXPB1. Only 12b induced symptoms when expressed from PVX in *N. benthamiana*, despite all four versions being expressed, as confirmed by western blotting (Fig. 2M). However, PVX-*Gr*EXPB2 did not induce any symptoms in *N. tabacum*, either by agroinfiltration or agroinfection, aside from the typical mosaic symptoms indicative of PVX infection (data not shown).

We next expressed *Gr*EXPB2 in eight tomato cultivars (LA1972, Bush Beefsteak, Yellow Pear-shaped, Cherry, Rose de Berne, Starfire, Earliana and Moneymaker) and four potato cultivars (Katahdin, Green Mountain, Hilite Russet, and Miranda) with PVX-*Gr*EXPB2. Potato cultivars Atlantic and Divina, which express the *Rx* gene, were inoculated with PVX-HB-*Gr*EXPB2. After agroinfection with PVX-*Gr*EXPB2, we observed a strong necrotic response around the inoculated site 7–10 days post inoculation (DPI) in all tomato and potato cultivars listed above. As in *N. benthamiana* the symptoms in tomato appeared only when the SP was included indicating that the phenotype is induced by *Gr*EXPB2 in the apoplast (Fig. 2F and 2G). To demonstrate that the necrotic phenotype is not affected by PVX we transiently expressed *Gr*EXPB2 from the binary construct, pEAQ35S in leaves of two tomato cultivars (LA1972 and Moneymaker). Cell death was induced in tomato by pEAQ35S-*Gr*EXPB2 at 3–4 DPI, similar to the response induced by expression of an autoactive mutant Rx (AtRx) protein [45] included as a control, while no symptoms were observed with the empty vector, *Gr*EXPB2ΔSP or several other effectors (Fig. 2H and 2I). PVX-*Gr*EXPB2 also induced strong necrotic symptoms at 10–15 DPI in agroinfected leaves in all potato cultivars tested (Fig. 2J), whereas no symptoms were observed in control sites inoculated with PVX-GFP, PVX-*Gr*PEL1 or PVX-*Gr*EXPB2ΔSP (Fig. 2J). Similar to the results obtained in *N. benthamiana*, only *Gr*EXPB2 clone 12b induced symptoms in tomato (Fig. 2K and 2L).

### Expression of *Gr*PEL1, *Gr*PEL2 and *Gr*MTP induces systemic chlorosis and necrosis

**Figure 3:**
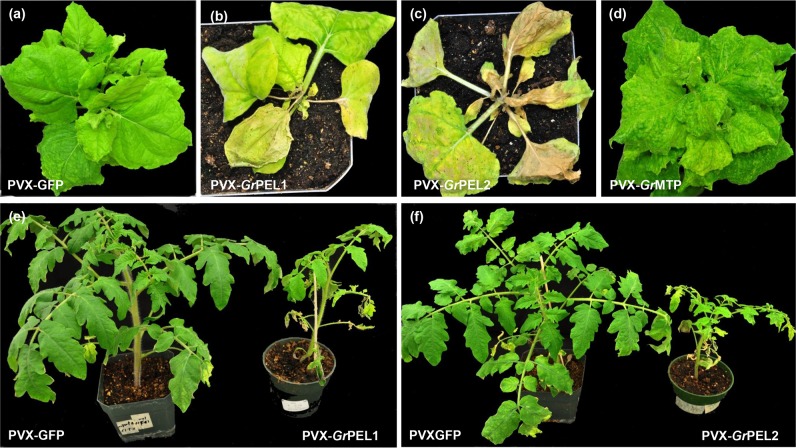
*Gr*PEL1, *Gr*PEL2 and *Gr*MTP induce chlorosis and necrosis in *N. benthamiana* and tomato. *N. benthamiana* plants were infected by agroinfiltration with (a) PVX-GFP, (b) PVX-*Gr*PEL1, (c) PVX-*Gr*PEL2, (d) PVX-*Gr*MTP. Plants were photographed at 21 DPI. Tomato plants, cultivar Starfire, were inoculated with (e) PVX-*Gr*PEL1 and (f) PVX-*Gr*PEL2 or with PVX-GFP (e, f, right hand side). Plants were photographed at 28 DPI.

Systemic expression of *Gr*PEL1, *Gr*PEL2 and *Gr*MTP in *N. benthamiana* resulted in severe malformations within the infiltrated leaves at six DPI. At fourteen DPI, PVX-*Gr*PEL1 induced severe curling, chlorosis and wrinkling in the upper leaves, eventually killing the plant at 21 DPI (Fig. 3B). PVX-*Gr*PEL2 did not induce severe chlorotic symptoms in the infiltrated leaves, but as it moved systemically it killed the midrib of the leaves, with necrosis spreading with virus movement, eventually killing the upper part of the plant as well as the inoculated leaves (Fig. 3C). *Gr*MTP induced malformation in the systemic leaves, with the leaves eventually becoming extremely discolored and cup shaped (Fig. 3D).

In tomato *Gr*PEL1 and *Gr*PEL2 induced systemic leaf crinkling and necrotic flecks, eventually killing some of the branches and resulting in the infected plants being significantly dwarfed (Fig. 3E and 3F).

### Expression of *Gr*CLE peptides produces dramatic developmental phenotypes

**Figure 4:**
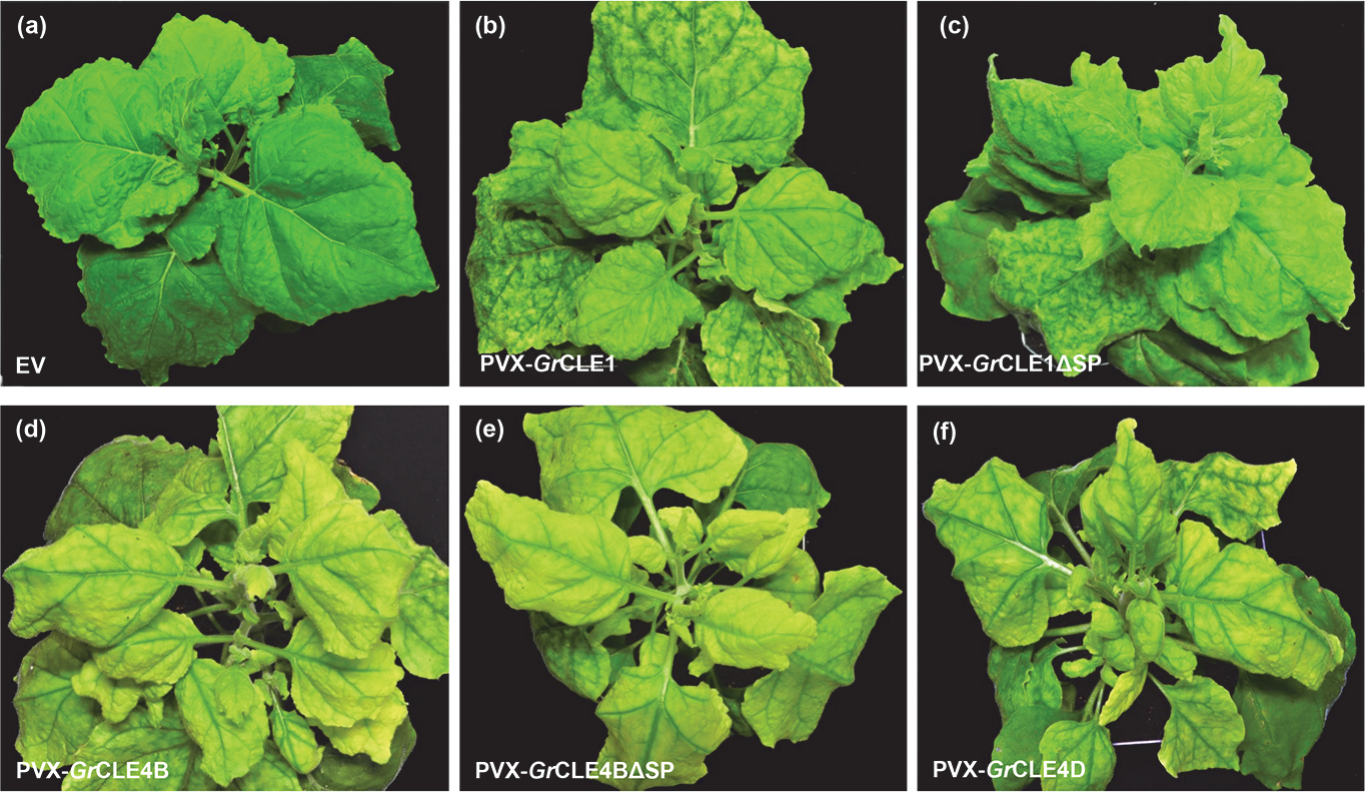
CLE peptides induce dramatic phenotypes in *N. benthamiana*. *N. benthamiana* plants were infected by agroinfiltration with (a) empty PVX vector (EV), (b) PVX-*Gr*CLE1, (c) PVX-*Gr*CLE1ΔSP, (d) PVX-*Gr*CLE4B, (e) PVX-*Gr*CLE4BΔSP and (f) PVX-*Gr*CLE4D. Photographs were taken at 21 DPI.

Five CLAVATA3/ESR-like (CLE) effectors (*Gr*CLE1, *Gr*CLE4D, *Gr*CLE4B, *Gr*CLE4C and *Gr*CLE4A) were expressed from PVX both with and without SP in *N. benthamiana, N. tabacum*, tomato and potato. *Gr*CLE1 induced downward curving leaves and chlorosis in the systemic leaves of infected *N. benthamiana* plants compared to the empty vector inoculated plant (Fig. 4A and 4B). A similar phenotype was observed both for the full-length *Gr*CLE1 construct as well as the *Gr*CLE1ΔSP construct lacking SP (Fig. 4B and 4C). *Gr*CLE4B, *Gr*CLE4BΔSP and *Gr*CLE4D induced severed chlorosis and downward curling in systemic leaves (Fig. 4D-F) with the leaves becoming very narrow and yellow. These plants also showed a significant increase in axillary shoots. The curved leaf phenotype was less pronounced in *Gr*CLE4C and no phenotype was observed with *Gr*CLE4A in *N. benthamiana* (data not shown). No unusual symptoms were observed with any of *Gr*CLE constructs in the other plant species tested.

### *Gr*UBCEP12 and *Gr*SKP1 affect plant morphology and may function in the apoplast

**Figure 5:**
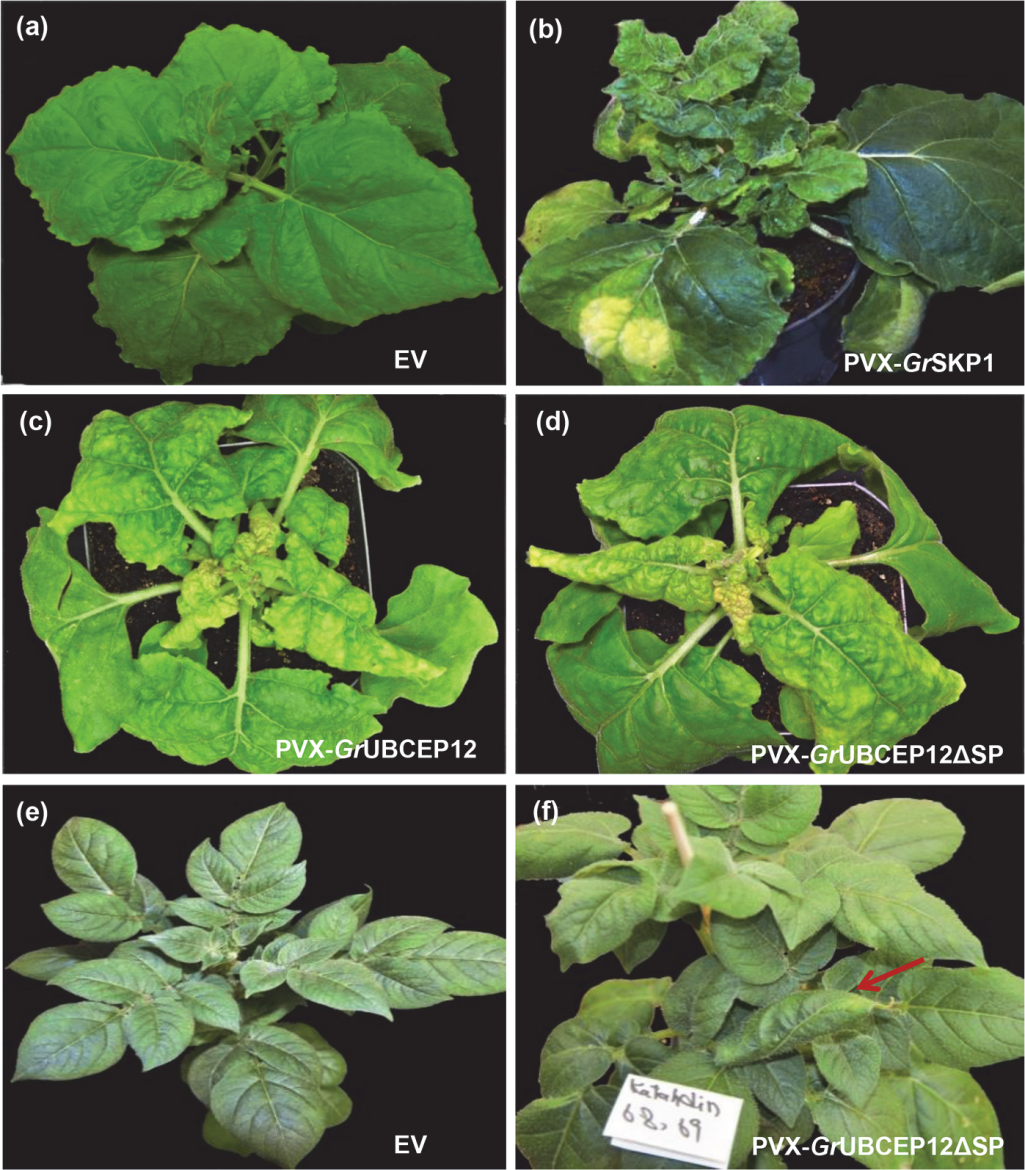
*Gr*UBCEP12 and *Gr*SKP1 alter plant morphology. *N. benthamiana* plants were infected by agroinfiltration with (a) empty PVX vector (EV), (b) PVX-*Gr*SKP1, (c) PVX-*Gr*UBCEP12, or (d) PVX-*Gr*UBCEP12ΔSP. Photographs were taken at 16 DPI. Systemic expression in potato of (e) PVX and (f) PVX-*Gr*UBCEP12ΔSP in potato cultivar Katahdin. Photographs were taken at 30 DPI.

The effectors *Gr*UBCEP12 and *Gr*SKP1 show striking homology to plant proteins involved in protein degradation. *Gr*SKP1 shows 75% identity to the Arabidopsis SKP1-related protein ASK10 (AT3G21860) over a 160 aa stretch whereas *Gr*UBCEP12 encodes a monoubiquitin domain and a small C-terminal carboxyl extension protein [32]. Although, intuitively, these proteins would be expected to function in the plant cytoplasm, we found that expressing the full-length proteins (including the SP) induced dramatic phenotypes. Expression of *Gr*SKP1 from PVX in *N. benthamiana* produced very bushy plants with an increased number of smaller leaves in the upper part of the plant. These leaves also presented a bubble-like phenotype on their upper surface compared to the empty vector inoculated plant (Fig. 5A and 5B). Expression of *Gr*UBCEP12 from PVX induced one of the most pronounced phenotypes in *N. benthamiana* and in potato. In *N. benthamiana* the phenotype first became visible 7–10 DPI, inducing shrunken downward curling leaves and eventually leading to the leaves turning brown and succumbing to a necrotic collapse after 16 DPI (Fig. 5D and 5D). A similar phenotype was observed with both full length *Gr*UBCEP12 and a version lacking the SP (*Gr*UBCEP12ΔSP) (Fig. 5C and 5D). PVX-*Gr*UBCEP12 was the only construct to induce a morphological phenotype in potato (cultivar Katahdin), where it induced leaf-curling symptoms (Fig. 5E and 5F).

### Suppression of immunity-associated cell death by *G. rostochiensis* effectors

**Figure 6:**
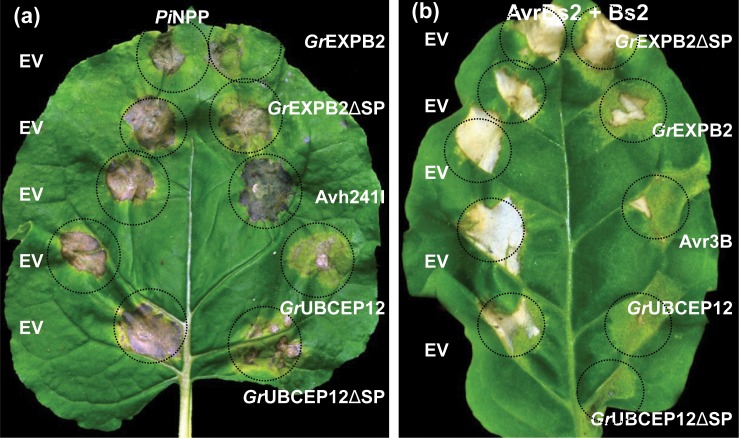
Suppression of cell death induced by NB-LRR proteins by apolastic effectors in *N. benthamiana* and *N. tabacum*. (a) *N. benthamiana* leaves were co-infiltrated with *Agrobacterium* carrying binary vectors expressing *Pi*NPP and P38 together with either empty vector (EV, left hand side) or the indicated effectors expressed from pEAQ35S (right hand side). (b) *N. tabacum* leaves were co-infiltrated with *Agrobacterium* containing binary vectors expressing AvrBS2, Bs2 and P38 together with either empty vector (EV, left hand side) or the indicated effectors expressed from pEAQ35S (right hand side). Cell death symptoms were assessed (Table 2) at 3–5 DPI and photographs were taken at 5 DPI.

We screened the CSEP constructs for their ability to suppress cell death. In the first screen, we transiently expressed effectors from PVX in the leaves of *N. benthamiana* and *N. tabacum* by agroinfiltration together with three cell death inducers, including the *P. infestans* elicitor PiNPP [46,47] as well as an autoactive mutant (D460V) of the NB-LRR Rx (AtRx) [28,45] and the combination of the NB-LRR Bs2 with its cognate effector AvrBs2 [48] (see Material and Methods). Assessment of cell death suppression activities is listed in Table 2 and representative assays are shown in Fig. 6. The cell death percentage scores were calculated from 12–15 infiltration sites based on three to four independent experiments. Two putative apoplastic effectors, *Gr*EXPB2 and *Gr*UBCEP12, inhibited the cell death induced by PiNPP, AtRX and AvrBs2/Bs2 in *N. benthamiana* and *N. tabacum* leaves (Table 2).

**Table 2:** Suppression of cell death by *G. rostochiensis* apoplastic effectors.

**Effector^a^**	**Suppression of cell death in *N. benthamiana*^b^**			**Suppression of cell death in *N. tabacum*^b^**		
	**AtRx**	***Pi*NPP**	**Bs2/AvrBs2**	**AtRx**	***Pi*NPP**	**Bs2/AvrBs2**
*Gr*CLE1	−	−	−	−	−	−
*Gr*CLE1ΔSP	−	−	−	−	−	−
*Gr*CLE-4A	−	−	−	−	−	−
*Gr*CLE-4D	−	−	−	−	−	−
*Gr*CLE-4DΔSP	−	−	−	−	−	−
*Gr*CLE-4B1	−	−	−	−	−	−
*Gr*CLE-4BΔSP	−	−	−	−	−	−
*Gr*CLE-4C	−	−	−	−	−	−
*Gr*CLE-4CΔSP	−	−	−	−	−	−
*Gr*EXPB1	−	−	−	−	−	−
*Gr*EXPB1ΔSP	−	−	−	−	−	−
*Gr*EXPB2	+	+++	+++	++	+	++
*Gr*EXPB2ΔSP	−	+	−	−	−	−
*Gr*VAP1	−	−	−	−	−	−
*Gr*AMS1	−	−	−	−	−	−
*Gr*AMS1ΔSP	−	−	−	−	−	−
*Gr*SXP1	−	−	−	−	−	−
*Gr*SXP1ΔSP	−	−	−	−	−	−
*Gr*A42	−	−	−	−	−	−
*Gr*UBCEP12	+	+	+	++	Nt	+
*Gr*UBCEP12ΔSP	++	+	++	+	Nt	++
*Gr*SKP1	−	−	−	−	−	−
*Gr*4D06	−	−	−	−	−	−
*Gr*4D06-ΔSP	−	−	−	−	−	−
*Gr*TPX	−	−	−	−	−	−
*Gr*ENG1	−	−	−	−	−	−
*Gr*ENG2	−	−	−	−	−	−
*Gr*ENG3	−	−	−	−	−	−
*Gr*GPX	−	−	−	−	−	−
*Gr*PEL1	−	−	−	−	−	−
*Gr*PEL2						
*Gr*MTP	−	−	−	−	−	−
*GrGPX2*	−	−	−	−	−	−
*Gr*CM-1-A	−	−	−	−	−	−
*P. infestans* Avr3a ΔSP	Nt	++	Nt	Nt	+	Nt
*P. sojae* Avr3b ΔSP	+	Nt	Nt	+	+	+
pGR106-empty	−	−	−	−	−	−

^a^All effectors were expressed from PVX based constructs.

^b^(+++) Cell death suppression in at least 75% of infiltrated patches; (++) Cell death suppression in at least 50% of infiltrated patches; (+) Cell death suppression in at least 25% of infiltrated patches; (−) No suppression or less than 25%; (Nt) Not tested.

The same effectors were also expressed in *N. benthamiana* and *N. tabacum* from pEAQ35S together with binary vectors expressing PiNPP and AvrBs2/Bs2. In *N. benthamiana* and *N. tabacum* the cell death induced by PiNPP and AvrBs2/Bs2 was suppressed by both *Gr*EXPB2 and *Gr*UBCEP12 (Fig. 6A and 6B).

### Suppression of disease resistance mediated by the NB-LRR proteins Rx and N

**Figure 7:**
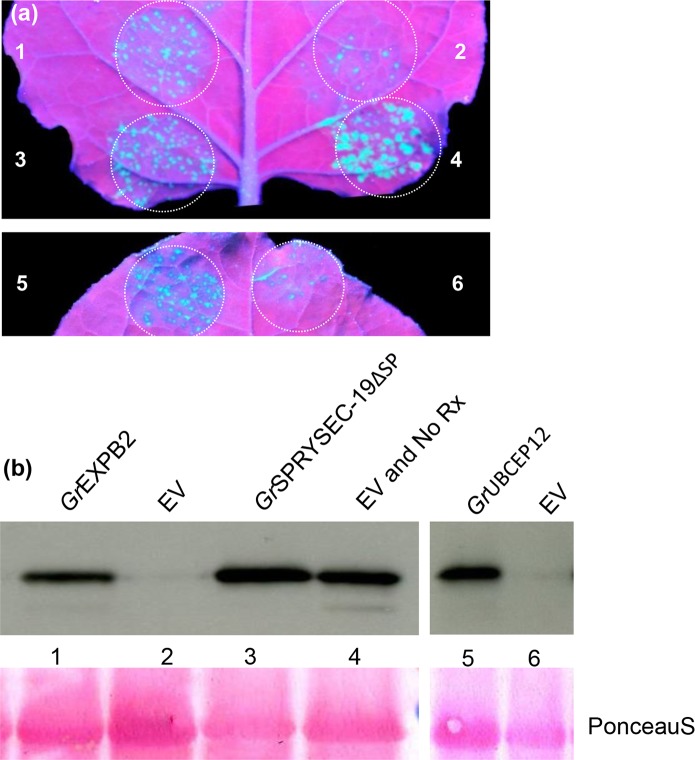
*Gr*EXPB2 and *Gr*UBCEP12 suppress Rx-mediated resistance to PVX. *N. benthamiana* leaves were co-infiltrated with *Agrobacterium* carrying binary vectors expressing PVX-GFP and 35S-Rx together with pEAQ35S vectors expressing 1, *Gr*EXPB2; 2, empty vector; 3, *Gr*SPRYSEC-19ΔSP; 4, Rx replaced with empty vector; 5, *Gr*UBCEP12; 6, empty vector. (a) GFP expression was visualized and photographed under UV illumination at 4 DPI. (b) Anti-GFP immune blotting was performed on total protein samples taken at 4 DPI from *N. benthamiana* patches expressing the same construct combinations as described above. Ponceau S staining (lower panel) was used to show equal loading.

**Figure 8:**
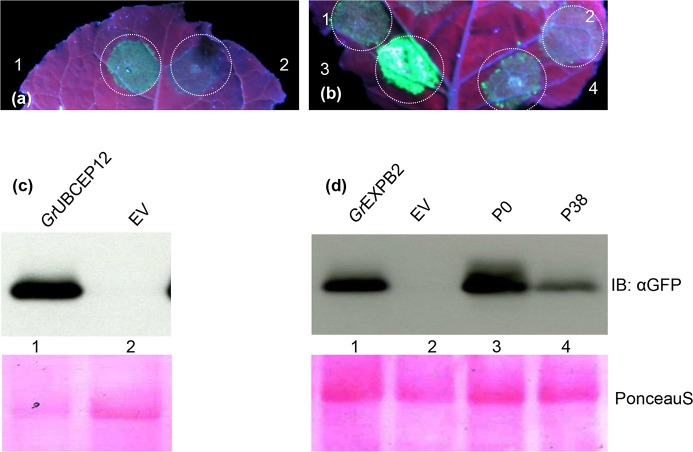
*Gr*EXPB2 and *Gr*UBCEP12 proteins suppress virus resistance mediated by the *N* gene. *N. benthamiana* leaves were co-infiltrated with *Agrobacterium* carrying binary vectors expressing PVX-GFP, N and P50 together with pEAQ35S expressing (a) 1, *Gr*UBCEP12; 2, empty vector; (b) 1, *Gr*EXPB2; 2, empty vector; 3, P0; 4, P38. GFP expression was visualized under UV illumination at 4 DPI. (c-d) Anti GFP immune blotting was performed on total protein samples taken at 4 DPI from *N. benthamiana* leaf patches co-expressing the combinations of constructs described in a and b. The number on the blot corresponds to the number on the leaf above each blot. Ponceau S staining (lower panel) was used to show equal loading.

Cell death is not required for preventing pathogen proliferation in plants in many cases, suggesting that additional mechanisms contribute to immunity. We investigated whether the putative apoplastic effectors that abrogate defense-related cell death could also suppress disease resistance mediated by the Rx and N proteins, which do not require the induction of cell death to confer resistance to viruses [30, 49]. In this assay, PVX expressing GFP (PVX-GFP) was agroexpressed in *N. benthamiana* leaves with the *Rx* gene along with either empty vector or the putative apoplastic effectors identified in the cell death suppression assays above. Virus accumulation was detected in *N. benthamiana* leaves by visualizing GFP by UV illumination and by immune-blotting at 4 DPI. Little or no GFP was observed in the leaf patches co-infiltrated with PVX-GFP, *Rx* and empty vector whereas both *Gr*EXPB2 and *Gr*UBCEP12 allowed significant accumulation of GFP in the infiltrated areas as assessed visually and by anti-GFP immune-blotting (Fig. 7). We also used an assay based on the *N* gene, which confers resistance to Tobacco mosaic virus (TMV) through the recognition of the P50 fragment of the viral replicase [30]. We have previously shown that co-expression of N and P50 in *N. benthamiana* leaves can inhibit the accumulation of an unrelated virus (PVX-GFP) in the absence of cell death [30]. Here, we co-expressed N and P50 plus PVX-GFP together with the two putative apoplastic defense-suppressing effectors and monitored the accumulation of PVX-GFP visually and by immune-blotting. The polerovirus P0 protein was included as a positive control in this assay as it has been shown to inhibit anti-viral defense responses, but not cell death [30]. As expected, P0 and *Gr*EXPB2 and *Gr*UBCEP12 inhibited the ability of N to suppress GFP accumulation in this assay (Fig. 8).

## Discussion

We have identified, cloned and partially characterized twenty-three putative CSEPs from *G. rostochiensis*. Given their activity when expressed with their SP, together with previous reports, at least fourteen of these are likely to function in the apoplast (Table 1). Surprisingly the *Gr*SKP1 and *Gr*UBCEP12 proteins, predicted to function in the cytoplasm, also induced effects when expressed with their SP, suggesting that these proteins may also function outside the cell. An additional seven proteins, which did not induce any effect *in planta*, and whose predicted functions do not suggest a likely site of action, remain to be characterized. While the phenotypes were observed in above ground tissues, *G. rostochiensis* is able to infect leaves [50] and as such it is reasonable to assume that its effectors will function similarly in these tissues, particularly for recognition by, and inhibition of, the immune system which likely functions similarly in all tissues.

Phytopathogenic nematodes encode at least three classes of effectors based broadly on their biological activities: effectors that degrade or modify host cell walls; effectors involved in reprogramming cellular identity and metabolism; and effectors involved in suppressing host defenses. Members of the first class, including several predicted cell wall degrading enzymes such as endoglucanases (*Gr*ENG1, *Gr*ENG2, *Gr*ENG3), pectate lyases (*Gr*PEL1, *Gr*PEL2) as well as expansin-like (*Gr*EXPB1, *Gr*EXPB2) proteins and a metalloproteinase (*Gr*MTP), would be expected to be apoplastic. Representative genes from this group (*GrPEL1, GrENG1* and *GrEXPB2*) showed relatively high expression in pre-J2s and during early stages of infection (S1 Fig.). The most severe phenotypes observed were those produced by over expression of *Gr*PEL1 and *Gr*PEL2 in *N. benthamiana* and tomato (Fig. 3). Despite showing only 28% identity and being predicted to target different pectic polysaccharides [51], both proteins induced severe necrosis. The phenotype that we observed with *Gr*PEL1 and *Gr*PEL2 expression (Fig. 3) is consistent with a role for pectate lyases in tissue maceration associated with numerous pathogens encoding pectate lyase activity [52]. In nematode infection, this phenotype would be expected to be highly localized, but would be beneficial to the nematode during its migration towards the target host cell.

The *Gr*CLE1 peptide has been shown to change root growth and morphology in Arabidopsis as well as in host plants potato and tomato [53] and *Gr*CLE4 peptides can rescue the Arabidopsis *clv3* mutant [34]. The morphological phenotypes induced by PVX-*Gr*CLE constructs (Fig. 4) are consistent with these nematode-secreted effector peptides mimicking endogenous plant CLE peptides and in reprogramming plant cell fate during syncytium formation and maintenance [14]. The fact that *Gr*CLE1 and *Gr*CLE4B can induce similar phenotypes with and without a SP is consistent with a report showing that mature propeptides of soybean cyst nematode CLE proteins are delivered first to the syncytium cytoplasm and then translocated to the apoplast by an unknown pathway [54,55]. Other candidate effectors that might be involved in reprogramming host cell identity and metabolism include *Gr*SKP1 and *Gr*UBCEP12. These two proteins induce the most striking morphological phenotypes when expressed from PVX (Fig. 5) and, given their strong similarity to proteins involved in protein turnover, it is plausible that they may alter multiple host pathways. Interestingly, *Gr*UBCEP12 induced a strong phenotype when expressed from PVX either with or without a signal peptide (Fig. 5C, 5D and 5F). *Gr*UBCEP12 has been shown to be processed into two functional units inside the cell, a free ubiquitin moiety and a 12-amino acid peptide from its carboxyl terminus [32]. It remains to be seen which of these moieties contribute to the observed phenotypes and whether they possess properties that allow them to exit (or enter) the cell by non-canonical mechanisms, as with the CLE peptides [54,55].

Although many cytoplasmic-delivered effectors have been shown to suppress the signaling pathways that lead to PTI [56], we have demonstrated that defense responses initiated from both the apoplast (PiNPP) and the cytoplasm (Rx, N) can be blocked by at least one apoplastic effector. Although the localizations of *Gr*UBCEP12 and *Gr*SKP1 remain to be definitively established, *Gr*EXPB2 appears to function in the apoplast given the fact that it does not function without its SP, consistent with its predicted function in binding cell wall-associated carbohydrates.

Expression of *Gr*EXPB2 is highly upregulated in the pre-J2 stage and diminishes quickly upon plant infection (S1 Fig.). *Gr*EXPB2 protein was also identified at high levels with several cell wall modifying enzymes and VAP1 in stylet secretions from pre-J2s indicating that these proteins are secreted into the apoplast to facilitate nematode invasion and migration, possibly through a cell wall-loosening activity [13]. The *G. rostochiensis* stylet can deliver apoplastic effectors, such as VAP1, ENG1 and EXPB2, in the preparasitic stage [13]. However cytoplasmic effectors can presumably only be delivered directly to the one cell the stylet eventually pierces. Thus it would make sense for the nematode to secrete apoplastic effectors that could suppress plant defenses during tissue invasion. *Gr*EXPB2 could be involved in suppression of early PTI and/or ETI during this stage. Other pathogens have been reported to suppress defenses from the apoplast. Pep1, an apoplastic effector from *Ustilago maydis*, suppresses cell death in maize by directly binding to cell wall-associated/apoplastic peroxidase [57]. Likewise, an apoplastic effector, calreticulin (Mi-CRT) from the root knot nematode *Meloidogyne incognita*, increases plant susceptibility to *M. incognita* and to an oomycete [58]. In addition, the extracellular growth-promoting peptide hormone phytosulfokine inhibits PTI in Arabidopsis [59]. These examples indicate that it is possible to suppress intracellular host defense signalling from the apoplast. However, this is, to our knowledge, the first example of an apoplastic effector that is able to inhibit NB-LRR responses.

Many effectors elicit defense responses, possibly because, in tampering with the host defense response, they inadvertently set off the response they were meant to defuse [60,61]. As such, one could predict that those effectors that suppress defense response might be the most likely to be recognized by the plant innate immune system. Indeed, the apoplastic *Gr*VAP1 protein interferes with protease-based defenses and in doing so elicits a resistance response from the Cf-2 protein [13]. Likewise, we suggest that *Gr*EXPB2 may also have the dual properties of suppressing and eliciting plant defenses.

*Gr*EXPB1 and *Gr*EXPB2 belong to an expansin-like family of proteins present in cyst, root-knot and migratory nematodes [42,43,62–65]. Proteins with expansin domains have also been reported in plant-parasitic bacteria and fungi [66,67]. In the saprophytic fungus *Trichoderma reesei* an expansin-like protein, the swollenin protein functions as a PAMP in *Trichoderma*-plant interaction by inducing local defense genes [67]. Homogenates from *G. rostochiensis* juveniles as well as extracts from leaves expressing *Gr*EXPB1 have been shown to induce plant cell wall extension [43]. Nonetheless, in contrast to *Gr*EXPB2 (type variant), *Gr*EXPB1 does not induce any phenotype in our *in planta* assays, nor does the *Gr*EXPB2 variant 7g or *Gp*EXPB2 (Fig. 2). As such, we suggest that the necrotic phenotypes induced by *Gr*EXPB2 are likely due to recognition by components of the plant innate immune system present in tomato and potato. This in turn would explain the fact that recombinant PVX expressing “avirulent” *Gr*EXPB2 is unable to infect tomato systemically (data not shown). Likewise, the fact that *Gr*EXPB2 appears to be recognized in potato and tomato, but not tobacco, plus the fact that the *Gr*EXPB2 (7g) and *Gp*EXPB2 are not recognized in tomato (Fig. 2) suggest specific recognition by the host immune system. We have modelled the 3D structure of *Gr*EXPB2 using the crystal structure of the EXPB1 protein (Zea m 1) [68] in the Swiss-Model Workspace [69,70] and found that all three amino acids that are altered in the two variant proteins were positioned on the outer surface of the protein (S4 Fig.). This suggests that these differences could affect interactions with other proteins, potentially including PRRs. As such, the chlorotic phenotype induced in *N. benthamiana* by PVX-EXPB2 may be due to a weak recognition of *Gr*EXPB2, whereas recognition would appear to be absent in tobacco. Defense induction by *Gr*EXPB2 is presumably mediated through transmembrane RLK or RLP proteins in tomato and potato. Why recognition of *Gr*EXPB2 does not confer resistance to *G. rostochiensis* in these plants is unclear. However, it may also be due to additional effectors that cooperate in suppressing defense responses as has been proposed for the interaction between *Gp*-RBP-1 and Gpa2 [16]. The nature of the interplay between *Gr*EXPB2’s role in suppressing and inducing plant defenses is a subject for future studies. Nonetheless, we have identified an important player in plant-nematode interactions, which may play a role in the often multi-genic mechanisms of defense against cyst nematodes.

## Supporting Information

S1 Fig Expression profile of four putative apoplastic CSEP encoding genes in different life stages of *Globodera rostochiensis*. The relative expression of four CSEP-encoding genes was determined using quantitative RT-PCR in five *G. rostochiensis* life stages: cyst, pre-parasitic second-stage juvenile (pre-J2) and parasitic second-, third- and fourth-stage juveniles (par-J2, J3 and J4). Values are means ± SE of two biological replicates, normalized to the *G. rostochiensis β-actin* gene and relative to expression in the egg stage.
(XLSX)

S2 Fig Sequence alignment of *Gr*EXPB1 and *Gr*EXPB2. CLUSTAL W2.1 (http://www.ebi.ac.uk/Tools/msa/clustalw2/) was used to align the amino acid sequences of *Gr*EXPB1 and *Gr*EXPB2. The signal peptide (SP; yellow highlighting) and Rare lipoprotein A (RlpA)-like double-psi beta-barrel (DPBB_1; grey highlighting) domains are conserved between the two proteins while carbohydrate binding domain (CDB_II; green highlighting) is absent from *Gr*EXPB2. Non identical residues or residues present only in one protein are represented by red coloring.
(PDF)

S3 Fig Sequence alignment of different EXPB2 clones from *G. rostochiensis* and *G. pallida*. (A) Nucleotide sequence alignment of three *G. rostochiensis*, EXPB2 clones; *GrExpB2* clone 1A, *GrExpB2* 12b and *GrExpB2* 7g and *G. pallida ExpB2, GpExpB2* clone 15l. Individual gDNA clones were obtained by PCR from gDNA isolated from cysts. Clones *GrExpB2* 7g and *GpExpB2* 15l were PCR amplified from gDNA and *GrExpB2* clone 1A, *GrExpB2* 12b were amplified from cDNA. The four introns are absent in *GpExpB2* clone 15l. Mismatched nucleotides are highlighted in red and identical nucleotides in yellow, the 3’ UTR is highlighted in green.
(B) Amino acid sequence alignment of *Gr*EXPB2 clone 1A, *Gr*EXPB2 12b, *Gr*EXPB2 7g and *Gp*EXPB2 clone 15l. The single mismatched amino acid between the two *Gr*EXPB2 clones is highlighted in pink and the two amino acids that differ from *Gp*EXPB2 are highlighted in red, all matched amino acids are highlighted in yellow.
(PDF)

S4 Fig 3D structure model of *Gr*EXPB2. ORFs from EXPB2 clones 12b (GenBank acc. no. GQ152150), 7g (GenBank acc. no. GQ152166) and 15l (GenBank acc. no. CAC84564.1) were used for 3D modelling with the Swiss-Model Workspace. Shown is the front and back view of the 3D structure model of *Gr*EXPB2 type protein (12b) as modelled on the crystal structure of the *Zea mays* protein EXPB1 (PDB ID: 2hczX). Residues variable between the type protein and variant clones are marked in colour: clone 7g (red) A65V; clone 15l (cyan) C79Y and L119M (green).
(PDF)

S5 Fig Nucleotide sequence alignment of fourteen *G. rostochiensis* effectors coding genes with reference genes. Multiple sequence alignment with hierarchical clustering (http://multalin.toulouse.inra.fr/multalin/multalin.html) was used for sequence alignment. Matched nucleotides are shown in red while the nucleotide that differ are shown either by blue or black and Genbank accession numbers are shown in parentheses. (A) Alignment of CLE-4A (top) with Reference CLE-4A. (B) Alignment of CLE-4B1 (top) with Reference CLE-4B1. (C) Alignment of ENG-1 (top) with Reference ENG-1. (D) Alignment of ENG-2 (bottom) with Reference ENG-2 (top). (E) Alignment of ENG-3 (top) with Reference ENG-3 (bottom). (F) Alignment of VAP1 (top) with Reference VAP1 (bottom). (G) Alignment of PEL1 (bottom) with Reference PEL1 (top). (H) Alignment of PEL2 (bottom) with Reference PEL2 (top). (I) Alignment of MTP (top) with Reference MTP (bottom). (J) Alignment of GPX2 (bottom) with Reference GPX (top). (K) Alignment of AMS1 (top) with Reference AMS1 (bottom). (L) Alignment of GPX1 (top) with Reference GPX1 (bottom). (M) Alignment of SKP1 (top) with Reference SKP1 (bottom). (N) Alignment of TPX (top) with Reference TPX (bottom).
(PPTX)

S1 Table Candidate effector proteins from *Globodera rostochiensis* with predicted Pfam domain, cysteine residues and its best matching sequences from other nematodes and (nr) nucleotide collection. (XLS)

S2 Table Sequence variation of candidate effector proteins coding genes from *Globodera rostochiensis*. (XLS)

S3 Table Primers used in this work (DOCX)
