# Supplementary material for: Analysis of Putative Apoplastic Effectors from the Nematode, Globodera rostochiensis, and Identification of an Expansin-Like Protein That Can Induce and Suppress Host Defenses
Source: PLoS One. 2015 Jan 21;10(1):e0115042. doi: 10.1371/journal.pone.0115042 (PMC4301866; doi:10.1371/journal.pone.0115042)
Supplement: S2 Fig — CLUSTAL W2.1 (http://www.ebi.ac.uk/Tools/msa/clustalw2/) was used to align the amino acid sequences of GrEXPB1 and GrEXPB2. The signal peptide (SP; yellow highlighting) and Rare lipoprotein A (RlpA)-like double-psi beta-barrel (DPBB_1; grey highlighting) domains are conserved between the two proteins while carbohydrate binding domain (CDB_II; green highlighting) is absent from GrEXPB2. Non identical residues or residues present only in one protein are represented by red coloring. (PDF) [file pone.0115042.s002.pdf]

```
                SP                                                   
GrEXPB1   MSSSEAILCLLCLLAVNFRAQIVLASVTAKLEGKSWNGGGQYVPNFKNNDGSKIACSVKF 60
GrEXPB2   MSCSQLILCLLCLLLVHPNES--------------------------------------- 16
          **.*: ******** *

                                 CBD_II
GrEXPB1   SLTPKKGTTIGSVWGANAVSGASNQYTLAPPADIAPGATHTNAGVNINGNGAPTLKLIEA 120
GrEXPB2   ------------------------------------------------------------ 20


GrEXPB1   KYFIDDVCGGAPAGSCMGCLSNTKMDGPINKNLNKPFKNSVFTFYGAGGRGACGLDAGVP 180
GrEXPB2   --------------CMGCLSSTTTDGPINQNLNKPFTNGVFTFNEATGRSACGLDAGKP 66
                        ******.*. *****:******.*.****   * **.******* *

GrEXPB1   KMSAAGSGNLFKPDGQWVDACRKDKRTLLDDPICKNICVKIDYNGKTLTVPINNKCPECT 240
GrEXPB2   KMSASVSGKLFKSDGQWKNACRIDQQYMLDDPICKNICVKIDYKGKSLTVPINNKCPECP 126
          ****: **:***.**** :*** *:: :**************:**:***********.
             DPBB_1
GrEXPB1   PSHVDLSIDAFNYLEPRGGLVGKATGX-----RSPI----- 271
GrEXPB2   PNNVDLSIDAFTYLESR--AVGKATGATLTYLKCPSGIKAC 165
```
