# Supplementary material for: Analysis of Putative Apoplastic Effectors from the Nematode, Globodera rostochiensis, and Identification of an Expansin-Like Protein That Can Induce and Suppress Host Defenses
Source: PLoS One. 2015 Jan 21;10(1):e0115042. doi: 10.1371/journal.pone.0115042 (PMC4301866; doi:10.1371/journal.pone.0115042)
Supplement: S3 Fig — (A) Nucleotide sequence alignment of three G. rostochiensis, EXPB2 clones; GrExpB2 clone 1A, GrExpB2 12b and GrExpB2 7g and G. pallida ExpB2, GpExpB2 clone 15l. Individual gDNA clones were obtained by PCR from gDNA isolated from cysts. Clones GrExpB2 7g and GpExpB2 15l were PCR amplified from gDNA and GrExpB2 clone 1A, GrExpB2 12b were amplified from cDNA. The four introns are absent in GpExpB2 clone 15l. Mismatched nucleotides are highlighted in red and identical nucleotides in yellow, the 3’ UTR is highlighted in green. (B) Amino acid sequence alignment of GrEXPB2 clone 1A, GrEXPB2 12b, GrEXPB2 7g and GpEXPB2 clone 15l. The single mismatched amino acid between the two GrEXPB2 clones is highlighted in pink and the two amino acids that differ from GpEXPB2 are highlighted in red, all matched amino acids are highlighted in yellow. (PDF) [file pone.0115042.s003.pdf]

```
GpExpB2-15l     ---------------------------------TGCCTTTTACTCGTCCATCCGAACGAA 27
GrExpB2-7g      ---------------------------------TGCCTTTTACTCGTCCATCCGAACGAA 27
GrExpB-12b      ---------------------------------TGCCTTTTACTCGTCCATCCGAACGAA 27
GrExpB2-1A      ATGAGCTGCTCCCAATTAATTCTGTGTTTGTTGTGCCTTTTACTCGTCCATCCGAACGAA 60
                                                 ***************************

GpExpB2-15l     TCTTG------------------------------------------------------- 32
GrExpB2-7g      TCTTGTAAAGATAAATATTAAATAAAATTTGATTTTTAATTTTTGGATCAATCCCTACCA 87
GrExpB2-12b     TCTTG------------------------------------------------------- 32
GrExpB2-1A      TCTTG------------------------------------------------------- 65
                *****

GpExpB2-15l     ------------------------------------------CATGGGCTGTCTGTCGAG 50
GrExpB2-7g      CAATGCCTATAACCCCTATATTATCATTCAATTTTCTAAAGGCATGGGCTGTCTGTCGAG 147
GrExpB2-12b     ------------------------------------------CATGGGCTGTCTGTCGAG 50
GrExpB2-1A      ------------------------------------------CATGGGCTGTCTGTCGAG 83
                                                          ******************

GpExpB2-15l     CACGACCACGGACGGGCCAATCAACCAGAACCTGAATAAGCCGTTCACGAACGGTGTCTT 110
GrExpB2-7g      CACGACCACGGACGGGCCAATCAACCAGAACCTGAATAAGCCGTTCACGAACGGTGTCTT 207
GrExpB2-12b     CACGACCACGGACGGGCCAATCAACCAGAACCTGAATAAGCCGTTCACGAACGGTGTCTT 110
GrExpB2-1A      CACGACCACGGACGGGCCAATCAACCAGAACCTGAATAAGCCGTTCACGAACGGTGTGTT 143
                ************************************************************

GpExpB2-15l     CACCTTCAACGAAGCCACTGGACGCAGCGCGTGCGGCCTTGACGCGGGCAAG-------- 162
GrExpB2-7g      CACCTTCAACGAAGCCACTGGACGCAGCGCGTGCGGCCTTGACGCGGGCAAGGTCAGTCA 267
GrExpB2-12b     CACCTTCAACGAAGCCACTGGACGCAGCGCGTGCGGCCTTGACGCGGGCAAG-------- 162
GrExpB2-1A      CACCTTCAACGAAGCCACTGGACGCAGCGCGTGCGGCCTTGACGCGGGCAAG-------- 195
                ****************************************************

GpExpB2-15l     ------------------------------------------------------------
GrExpB2-7g      AGAGTCTCGTTCTGACTATAAAAAAATTGCGCACAATTGGATCCTGATCAGTTAGACGTT 327
GrExpB2-12b     ------------------------------------------------------------
GrExpB2-1A      ------------------------------------------------------------

GpExpB2-15l     ------------------------------------------------------------
GrExpB2-7g      AAAATTCGCACTAAATTGATCAGGTGTTCAATTATGAACTCAACTGTCCTGTTTATGTCT 387
GrExpB2-12b     ------------------------------------------------------------
GrExpB2-1A      ------------------------------------------------------------

GpExpB2-15l     -------------------CCCAAAATGTCGGCATCCGTATCCGGCAAACTGTTCAAGTC 203
GrExpB2-7g      TACTGAAAAATTCAATTAGCCCAAAATGTCGGCATCCGTATCCGGCAAACTGTTCAAGTC 447
GrExpB2-12b     -------------------CCCAAAATGTCGGCATCCGTATCCGGCAAACTGTTCAAGTC 203
GrExpB2-1A      -------------------CCCAAAATGTCGGCATCCGTATCCGGCAAACTGTTCAAGTC 236
                                   *****************************************

GpExpB2-15l     GGACGGGCAATGGAAGAACGCGTGCCGGATAGACCAGCAGTACATGTTGGACGACCCGAT 263
GrExpB2-7g      GGACGGGCAATGGAAGAACGTGTGCCGGATAGACCAGCAGTACATGTTGGACGACCCGAT 507
GrExpB2-12b     GGACGGGCAATGGAAGAACGCGTGCCGGATAGACCAGCAGTACATGTTGGACGACCCGAT 263
GrExpB2-1A      GGACGGGCAATGGAAGAACGCGTGCCGGATAGACCAGCAGTACATGTTGGACGACCCGAT 296
                ******************** ***************************************

GpExpB2-15l     TTACAAGAACATTTGCGTGAAGATTGACTACAAAGGGAAGAG------------------ 305
GrExpB2-7g      TTGCAAGAACATTTGCGTGAAGATTGACTACAAAGGGAAGAGGTGCGCAAATTTGTTTGG 567
GrExpB2-12b     TTGCAAGAACATTTGCGTGAAGATTGACTACAAAGGGAAGAG------------------ 305
GrExpB2-1A      TTGCAAGAACATTTGCGTGAAGATTGACTACAAAGGGAAGAG------------------ 338
                ** ***************************************
```

```
GpExpB2-15l     -------------------------------------TCTGACCGTGCCGATCAACAACA 328
GrExpB2-7g      ATATTTTAACCTAATCAAAAGTTGCAACCCCTTGAAGTCTGACCGTGCCGATCAACAACA 627
GrExpB2-12b     -------------------------------------TCTGACCGTGCCGATCAACAACA 328
GrExpB2-1A      -------------------------------------TCTGACCGTGCCGATCAACAACA 361
                                                     ***********************

GpExpB2-15l     AGTGCCCGGAATGTCCGCCTAACAATGTGGATTTGTCGATTGACGCCTTCACCTATATGG 388
GrExpB2-7g      AGTGCCCGGAATGTCCGCCTAACAATGTGGATTTGTCGATTGACGCCTTCACCTATTTGG 687
GrExpB2-12b     AGTGCCCGGAATGTCCGCCTAACAATGTGGATTTGTCGATTGACGCCTTCACCTATTTGG 388
GrExpB2-1A      AGTGCCCGGAATGTCCGCCTAACAATGTGGATTTGTCGATTGACGCCTTCACCTATTTGG 421
                ******************************************************** ***

GpExpB2-15l     AGTCCCGTGCTGTGGGCAAAGCAACGGG-------------------------------- 416
GrExpB2-7g      AGTCCCGTGCTGTGGGCAAAGCAACGGGTTAGAATTACATATGTCGTTATGATTGATTTA 747
GrExpB2-12b     AGTCCCGTGCTGTGGGCAAAGCAACGGG-------------------------------- 416
GrExpB2-1A      AGTCCCGTGCTGTGGGCAAAGCAACGGG-------------------------------- 449
                ****************************

GpExpB2-15l     ------------------------------------------------------------
GrExpB2-7g      TACGGGGAGGGGGGCGTAAATAATAATCCGGGACTCGTCGTCAAGACAAAAAAAAATAGC 807
GrExpB2-12b     ------------------------------------------------------------
GrExpB2-1A      ------------------------------------------------------------

GpExpB2-15l     ------------------------------------------------------------
GrExpB2-7g      ACCGGTATCGGCAGGACTAAAAGCTTAGGATTTTCTAACCTTCCGGGCTGGGGATTTGTT 867
GrExpB2-12b     ------------------------------------------------------------
GrExpB2-1A      ------------------------------------------------------------

GpExpB2-15l     ------------------------------------------------------------
GrExpB2-7g      TAGTGATCAAAGAGCCTAAATTTATTGAATTTTAATCGATCAAGACAATATGCCGGCATT 927
GrExpB2-12b     ------------------------------------------------------------
GrExpB2-1A      ------------------------------------------------------------

GpExpB2-15l     ------------------------------------------------------------
GrExpB2-7g      TCTCTGTATTTAATTTTTTACAAATTTATATCAGATTTAATTGTCTTAATTAAGAAAAGT 987
GrExpB2-12b     ------------------------------------------------------------
GrExpB2-1A      ------------------------------------------------------------

GpExpB2-15l     -----------------------CGCTACGCTCACCTATTTAAAATGTCCGTCGGGCATC 453
GrExpB2-7g      GATGAAATACAAATTATTTTAGGCGCTACGCTCACCTATTTAAAATGTCCGTCGGGCATC 1047
GrExpB2-12b     -----------------------CGCTACGCTCACCTATTTAAAATGTCCGTCGGGCATC 453
GrExpB2-1A      -----------------------CGCTACGCTCACCTATTTAAAATGTCCGTCGGGCATC 486
                                       *************************************

GpExpB2-15l     AAAGCTTGCTGAAAATTTGGGGGCGTGAAAAATTTGACAATGACAAGTCGATCAATTCAT 513
GrExpB2-7g      AAAGCTTGCTGAAAATTTGGGGGCGTGAAAAATTTGACAATGACAAGTCGATCAATTCAT 1107
GrExpB2-12b     AAAGCTTGCTGAAAATTTGGGGGCGTGAAAAATTTGACAATGACAAGTCGATCAATTCAT 513
GrExpB2-1A      AAAGCTTGCTGA------------------------------------------------ 498
                ************

GpExpB2-15l     TATTTTATTGTCTAGCCAATTTATTCGACA 543
GrExpB2-7g      TATTTTATTGTCTAGCCAATTTATTCGACA 1137
GrExpB2-12b     TATTTTATTGTCTAGCCAATTTATTCGACA 543
GrExpB2-1A      ------------------------------
```

Figure S3A:

```
GrEXPB2-7g      -----------CLLLVHPNESCMGCLSSTTTDGPINQNLNKPFTNGVFTFNEATGRSACG 49
GrEXPB2-12b     -----------CLLLVHPNESCMGCLSSTTTDGPINQNLNKPFTNGVFTFNEATGRSACG 49
GrEXPB2-1A      MSCSQLILCLLCLLLVHPNESCMGCLSSTTTDGPINQNLNKPFTNGVFTFNEATGRSACG 60
GpEXPB2-15l     -----------CLLLVHPNESCMGCLSSTTTDGPINQNLNKPFTNGVFTFNEATGRSACG 49
                           *************************************************

GrEXPB2-7g      LDAGKPKMSASVSGKLFKSDGQWKNVCRIDQQYMLDDPICKNICVKIDYKGKSLTVPINN 109
GrEXPB2-12b     LDAGKPKMSASVSGKLFKSDGQWKNACRIDQQYMLDDPICKNICVKIDYKGKSLTVPINN 109
GrEXPB2-1A      LDAGKPKMSASVSGKLFKSDGQWKNACRIDQQYMLDDPICKNICVKIDYKGKSLTVPINN 120
GpEXPB2-15l     LDAGKPKMSASVSGKLFKSDGQWKNACRIDQQYMLDDPIYKNICVKIDYKGKSLTVPINN 109
                *************************.************* ********************

GrEXPB2-7g      KCPECPPNNVDLSIDAFTYLESRAVGKATGATLTYLKCPSGIKAC 154
GrEXPB2-12b     KCPECPPNNVDLSIDAFTYLESRAVGKATGATLTYLKCPSGIKAC 154
GrEXPB2-1A      KCPECPPNNVDLSIDAFTYLESRAVGKATGATLTYLKCPSGIKAC 165
GpEXPB2-15l     KCPECPPNNVDLSIDAFTYMESRAVGKATGATLTYLKCPSGIKAC 154
                *******************:*************************
```

Figure S3 B
