# Supplementary figures and images for: Analysis of Putative Apoplastic Effectors from the Nematode, Globodera rostochiensis, and Identification of an Expansin-Like Protein That Can Induce and Suppress Host Defenses
Source: PLoS One. 2015 Jan 21;10(1):e0115042. doi: 10.1371/journal.pone.0115042 (PMC4301866; doi:10.1371/journal.pone.0115042)

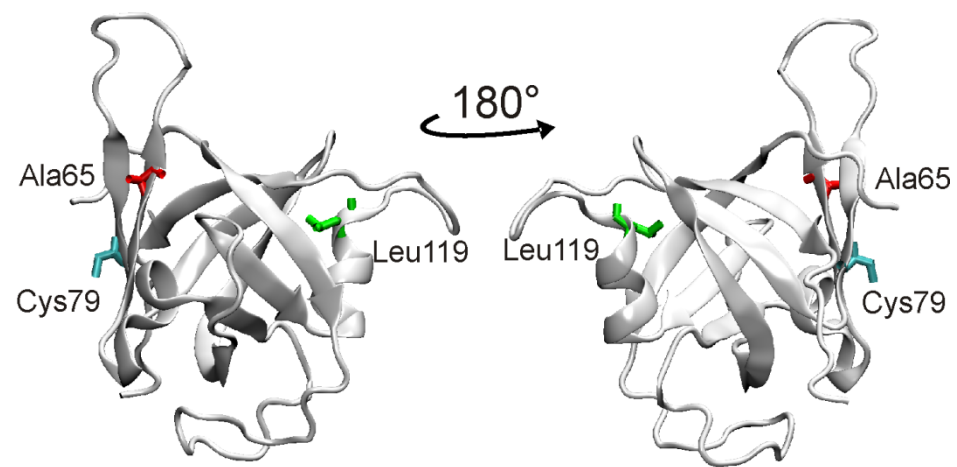

Supplement: S4 Fig — ORFs from EXPB2 clones 12b (GenBank acc. no. GQ152150), 7g (GenBank acc. no. GQ152166) and 15l (GenBank acc. no. CAC84564.1) were used for 3D modelling with the Swiss-Model Workspace. Shown is the front and back view of the 3D structure model of GrEXPB2 type protein (12b) as modelled on the crystal structure of the Zea mays protein EXPB1 (PDB ID: 2hczX). Residues variable between the type protein and variant clones are marked in colour: clone 7g (red) A65V; clone 15l (cyan) C79Y and L119M (green). (PDF) [file pone.0115042.s004.pdf]
