# Supplementary material for: Analysis of Putative Apoplastic Effectors from the Nematode, Globodera rostochiensis, and Identification of an Expansin-Like Protein That Can Induce and Suppress Host Defenses
Source: PLoS One. 2015 Jan 21;10(1):e0115042. doi: 10.1371/journal.pone.0115042 (PMC4301866; doi:10.1371/journal.pone.0115042)
Supplement: S5 Fig — Multiple sequence alignment with hierarchical clustering (http://multalin.toulouse.inra.fr/multalin/multalin.html) was used for sequence alignment. Matched nucleotides are shown in red while the nucleotide that differ are shown either by blue or black and Genbank accession numbers are shown in parentheses. (A) Alignment of CLE-4A (top) with Reference CLE-4A. (B) Alignment of CLE-4B1 (top) with Reference CLE-4B1. (C) Alignment of ENG-1 (top) with Reference ENG-1. (D) Alignment of ENG-2 (bottom) with Reference ENG-2 (top). (E) Alignment of ENG-3 (top) with Reference ENG-3 (bottom). (F) Alignment of VAP1 (top) with Reference VAP1 (bottom). (G) Alignment of PEL1 (bottom) with Reference PEL1 (top). (H) Alignment of PEL2 (bottom) with Reference PEL2 (top). (I) Alignment of MTP (top) with Reference MTP (bottom). (J) Alignment of GPX2 (bottom) with Reference GPX (top). (K) Alignment of AMS1 (top) with Reference AMS1 (bottom). (L) Alignment of GPX1 (top) with Reference GPX1 (bottom). (M) Alignment of SKP1 (top) with Reference SKP1 (bottom). (N) Alignment of TPX (top) with Reference TPX (bottom). (PPTX) [file pone.0115042.s005.pptx]

## Slide 1
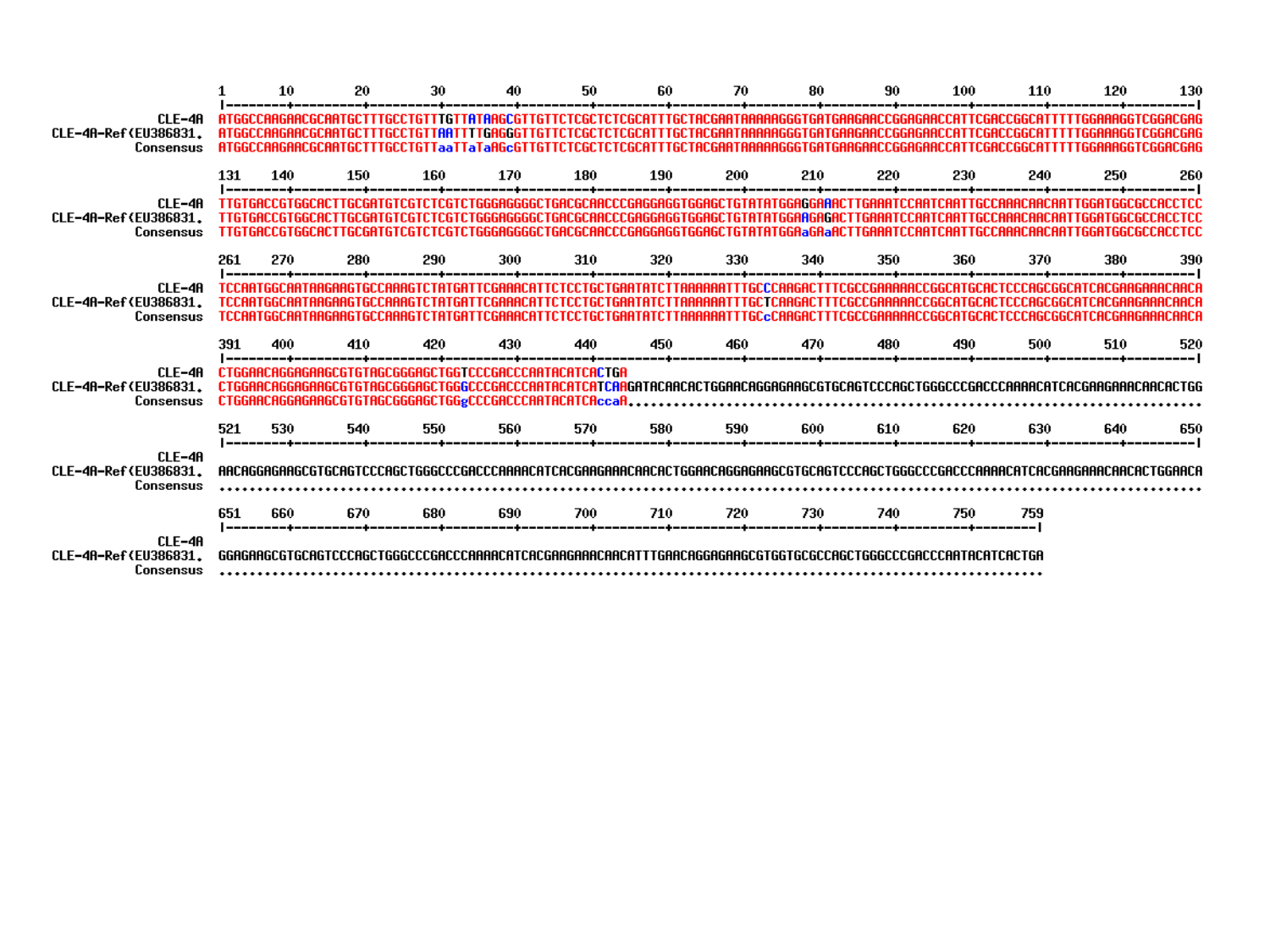

## Slide 2
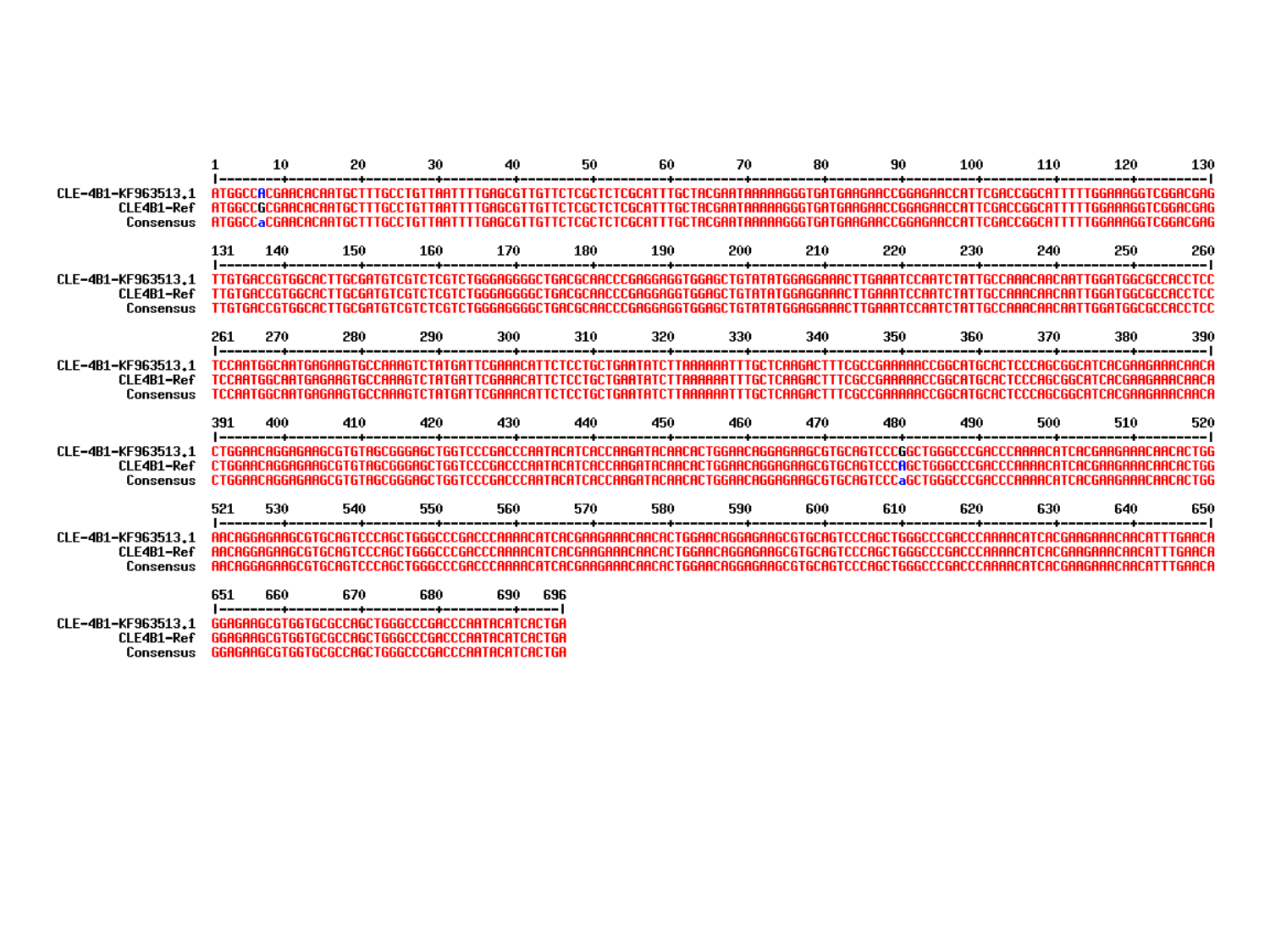

## Slide 3
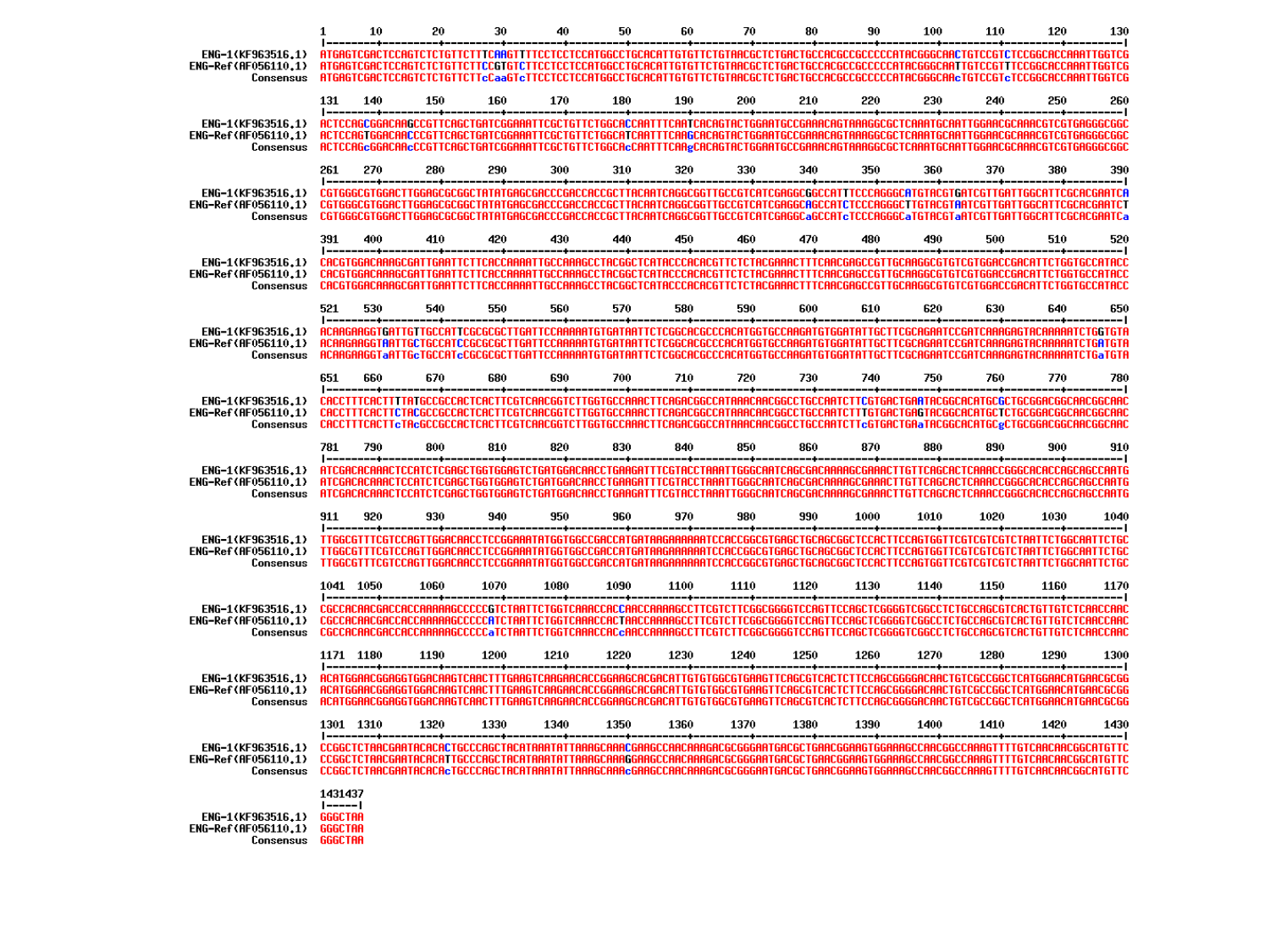

## Slide 4
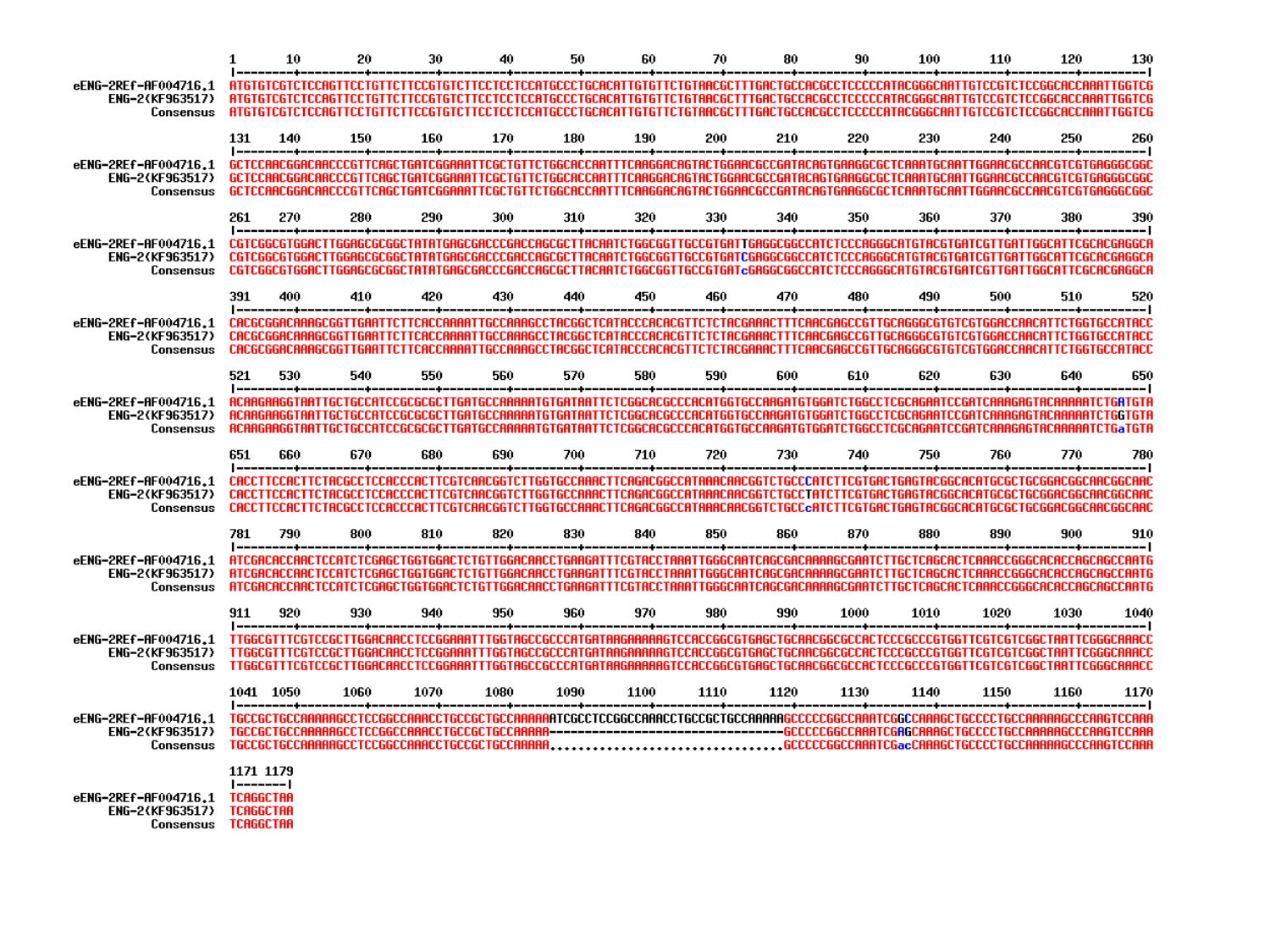

## Slide 5
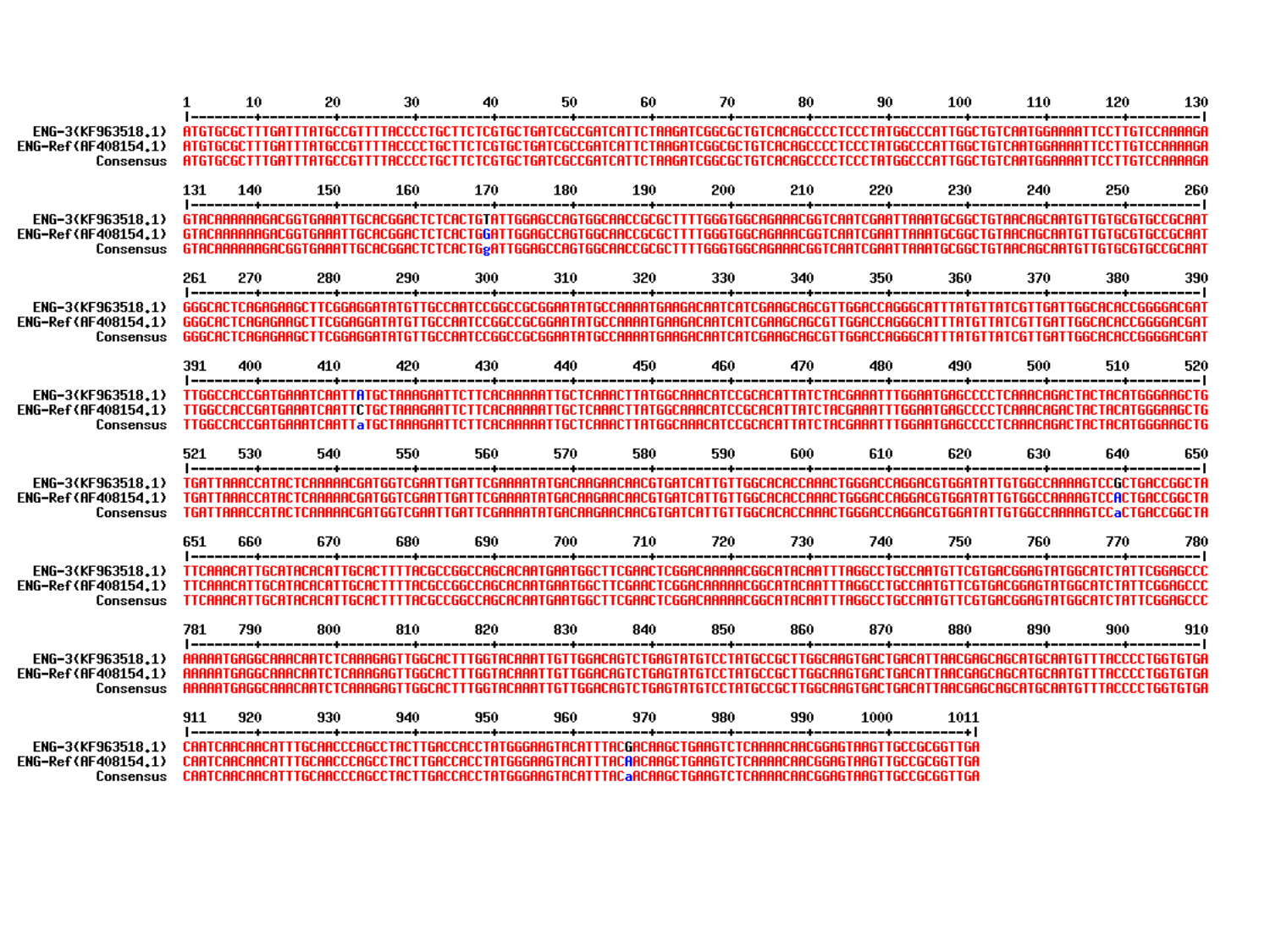

## Slide 6
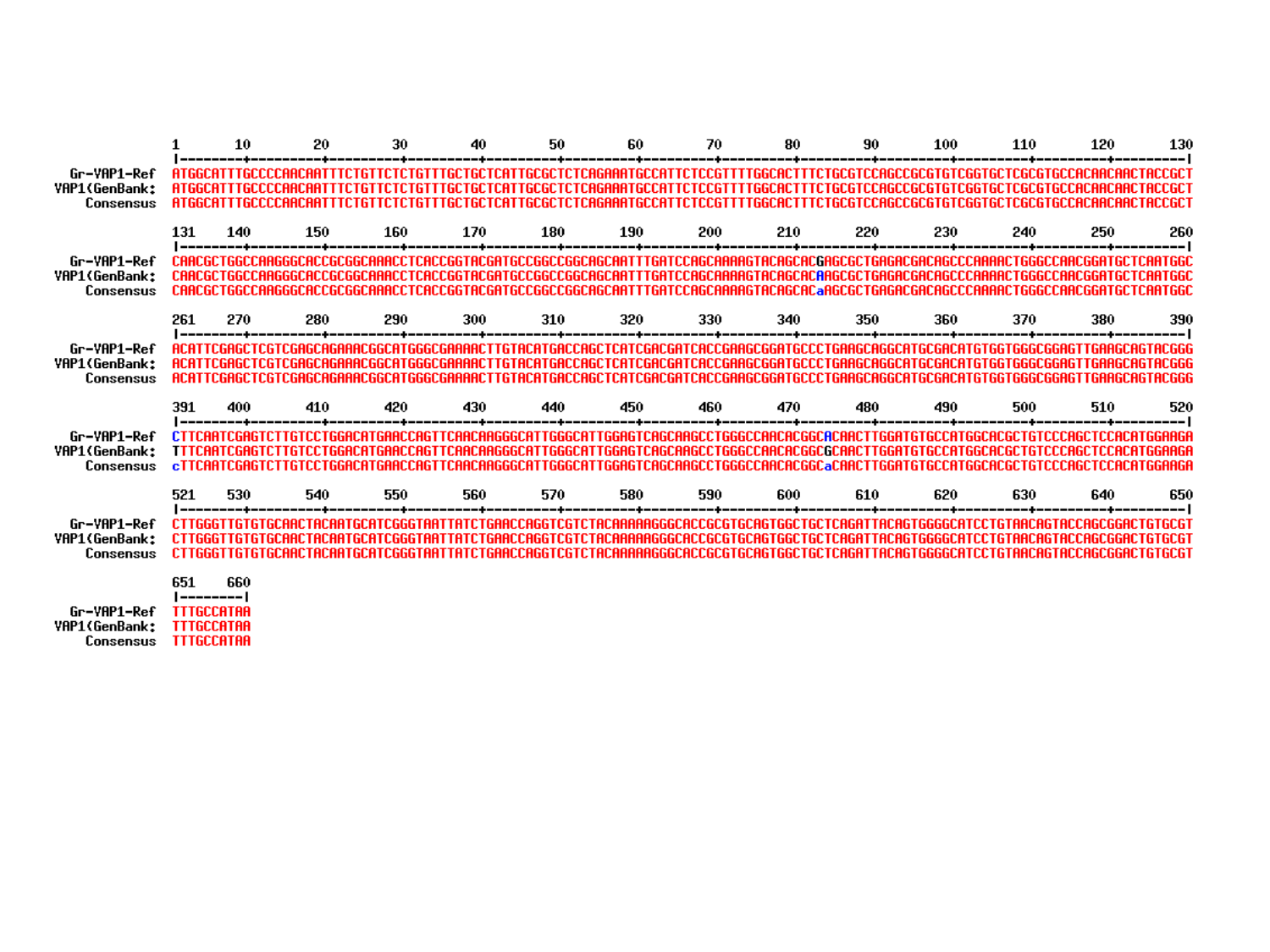

## Slide 7
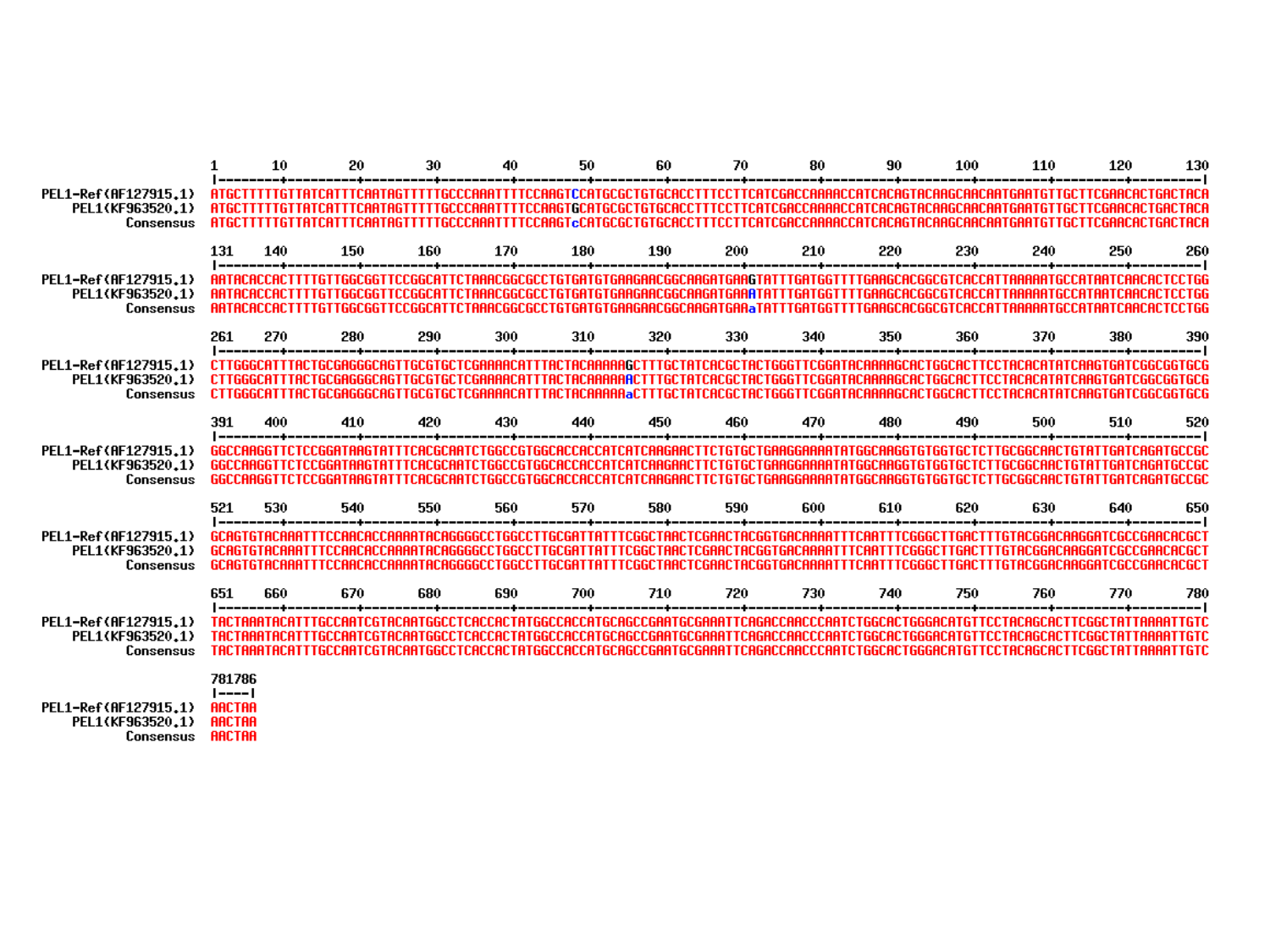

## Slide 8
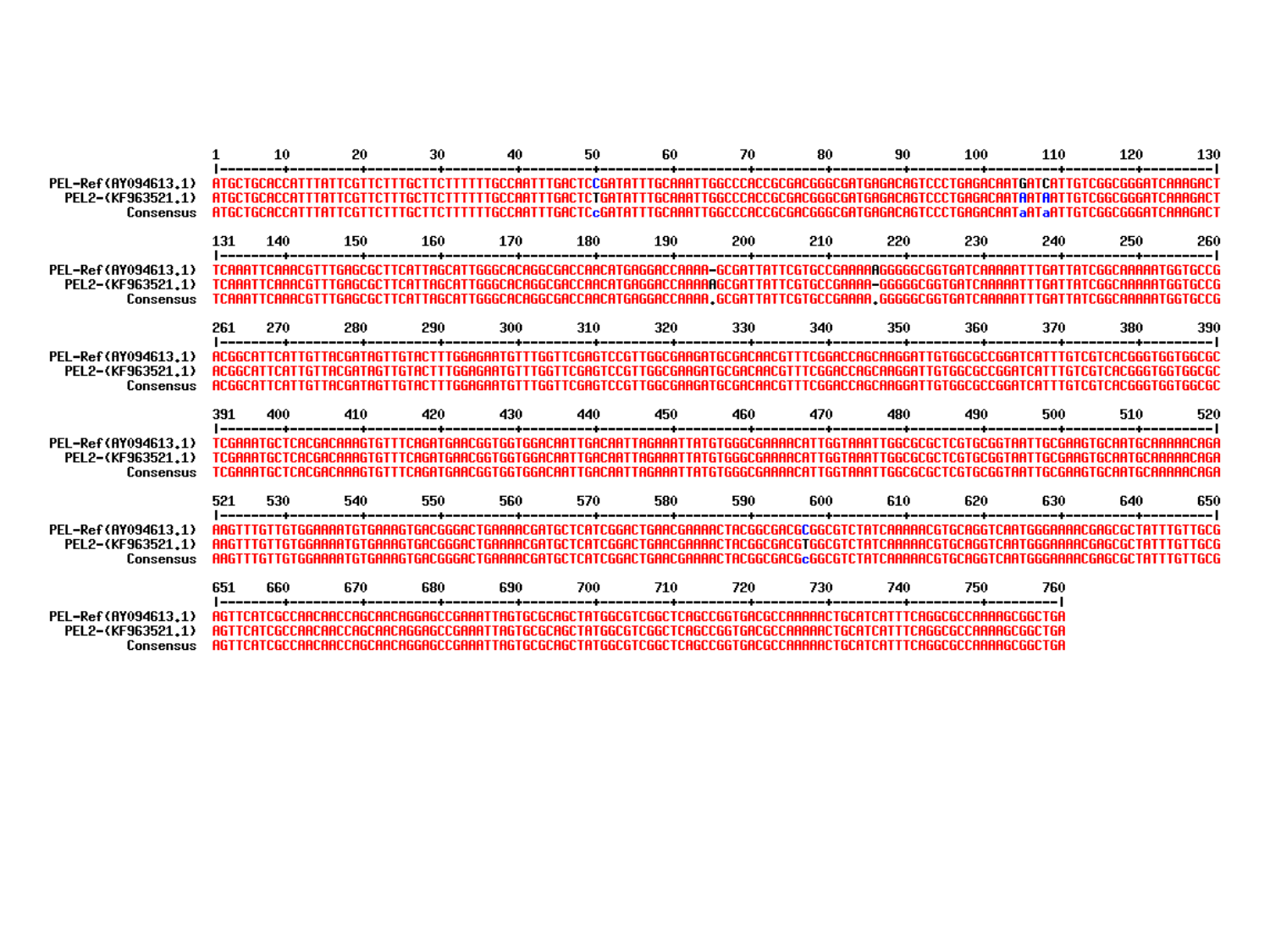

## Slide 9
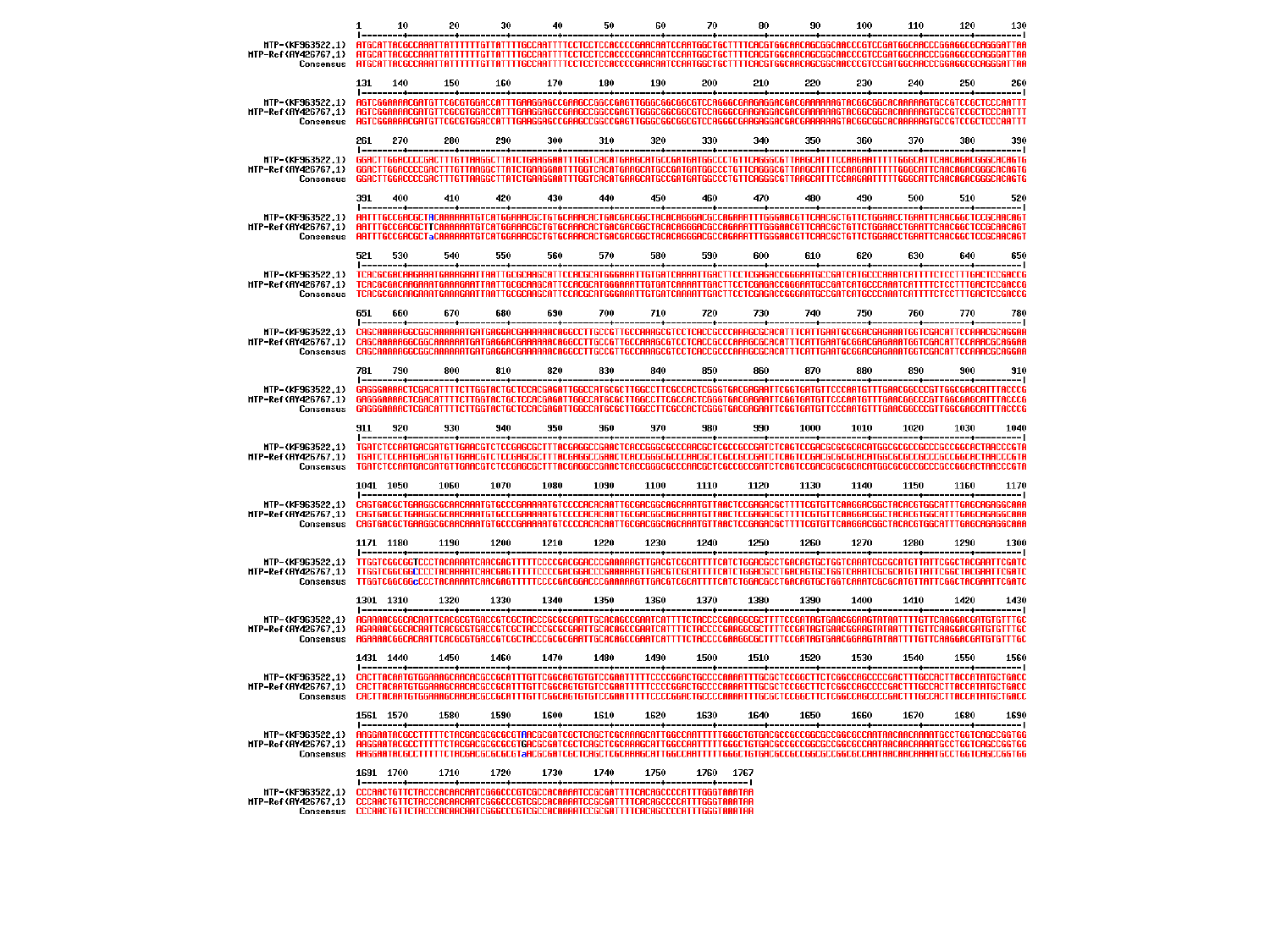

## Slide 10
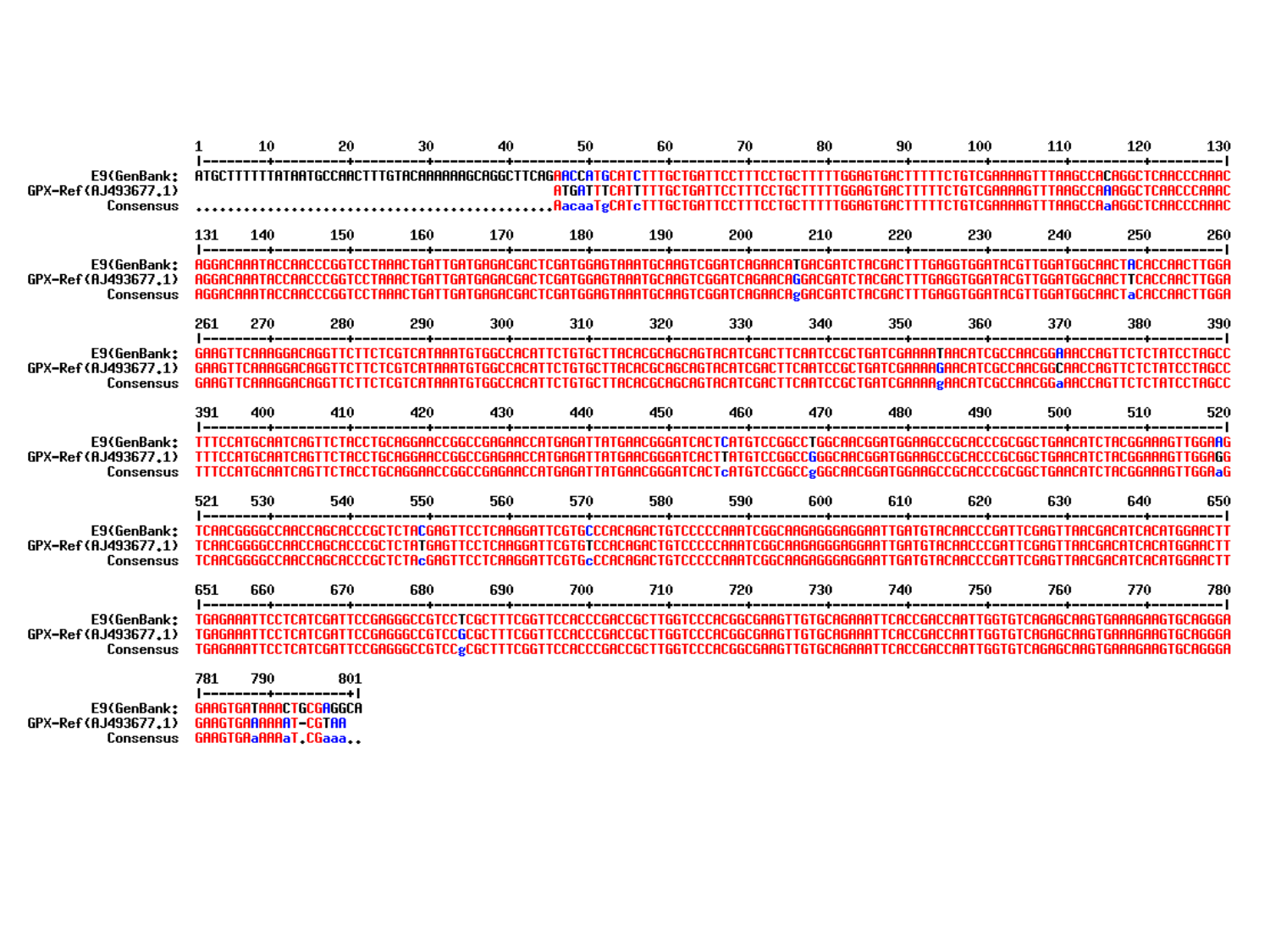

## Slide 11
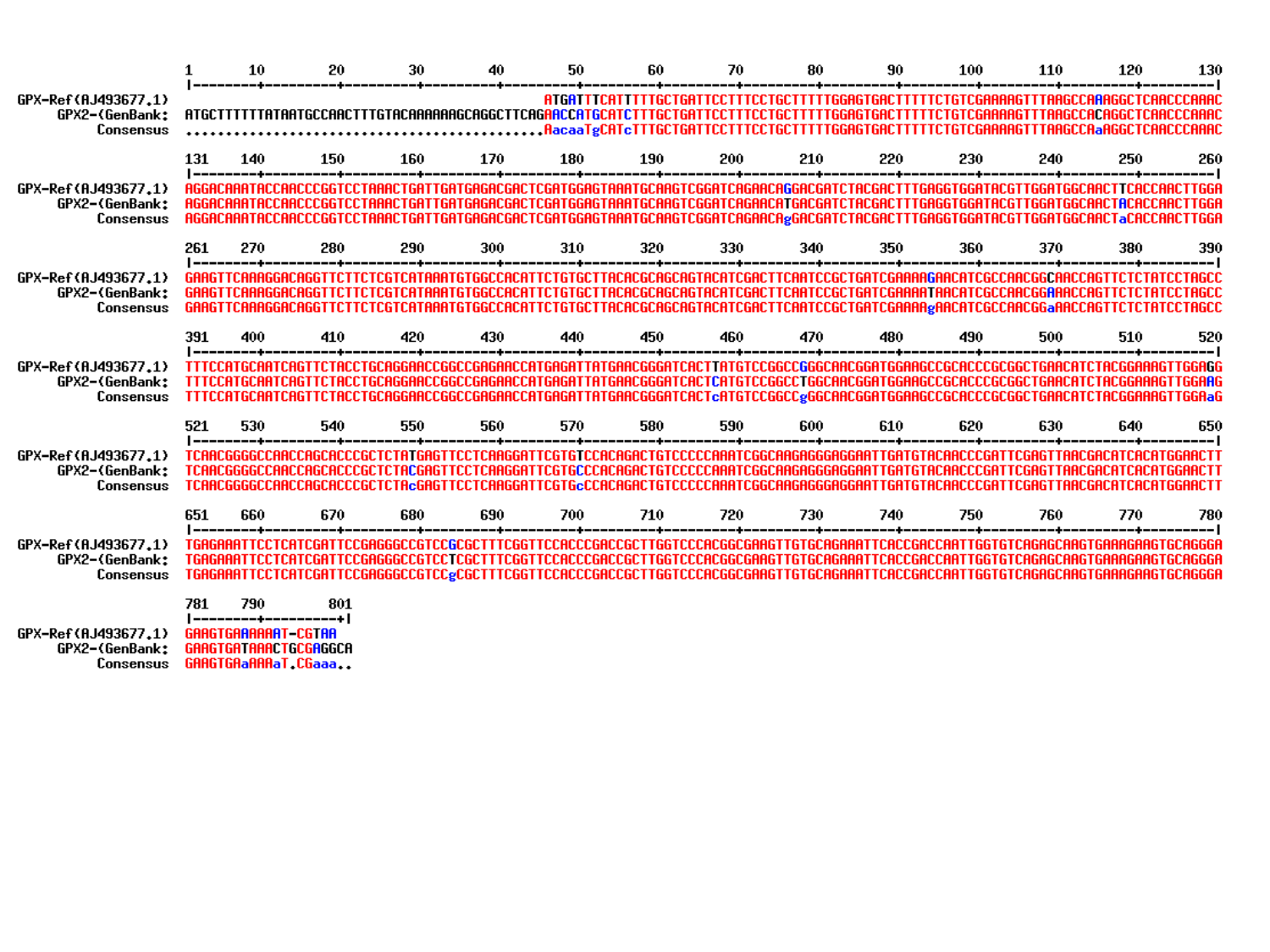

## Slide 12
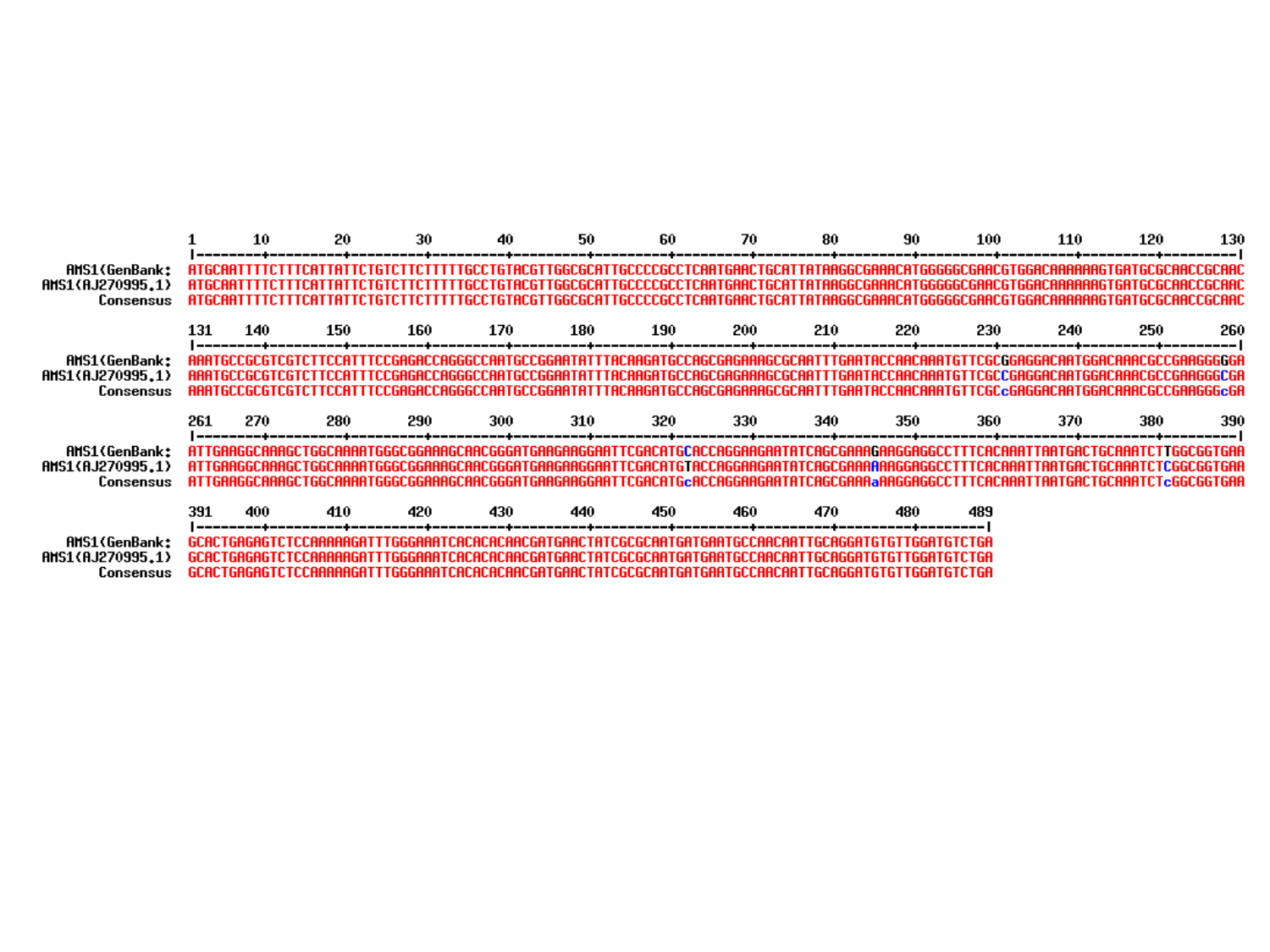

## Slide 13
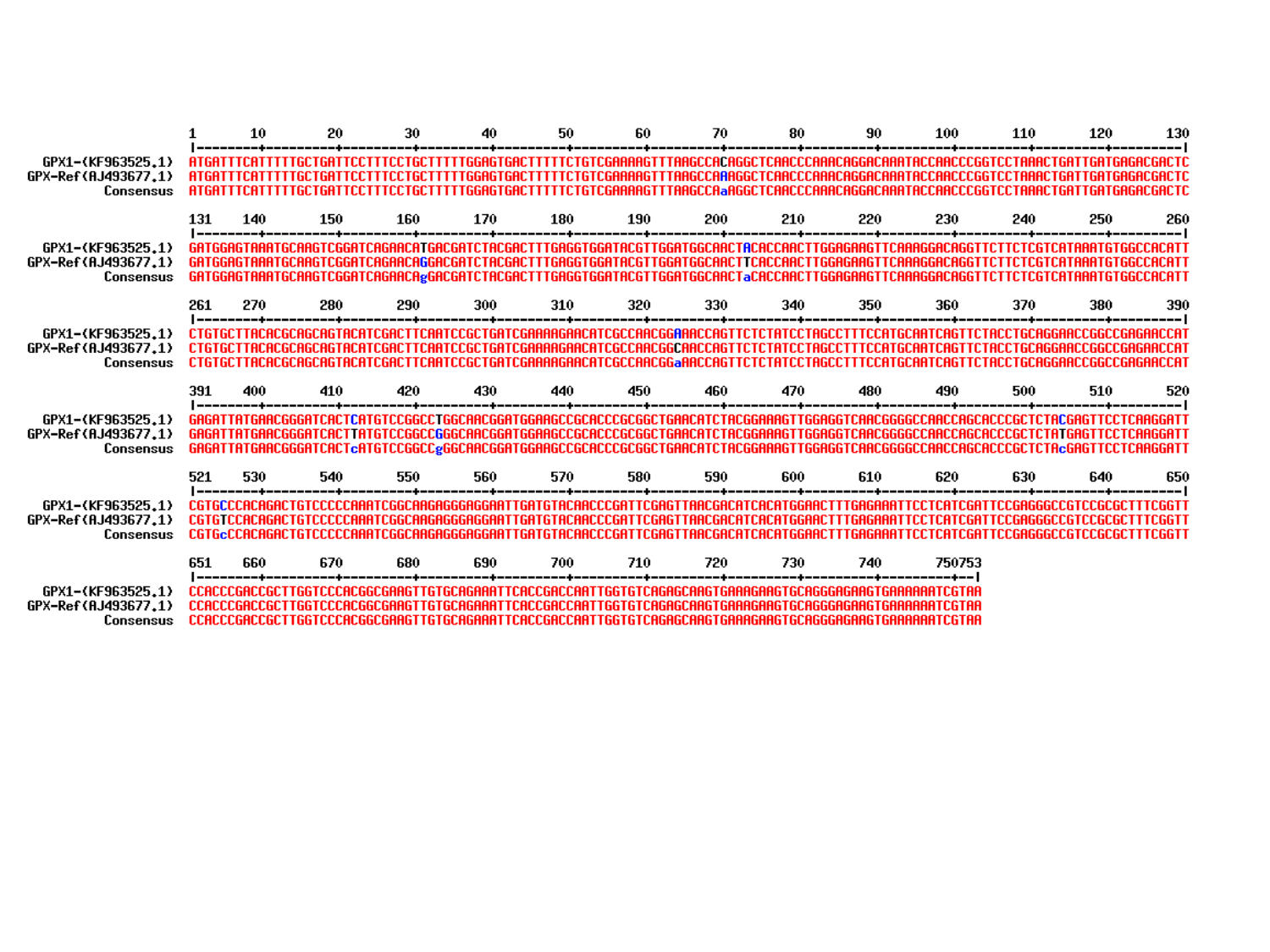

## Slide 14
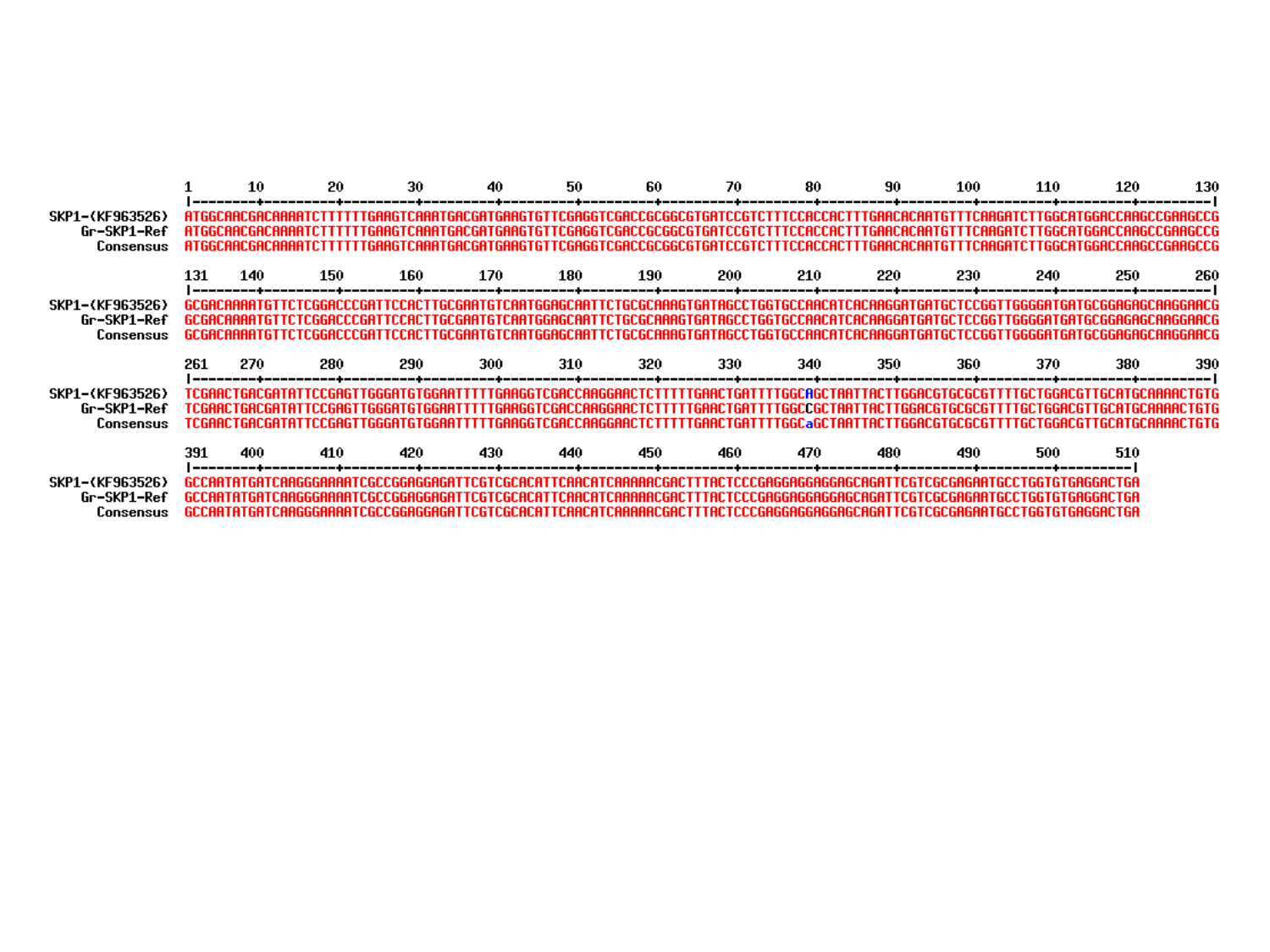

## Slide 15
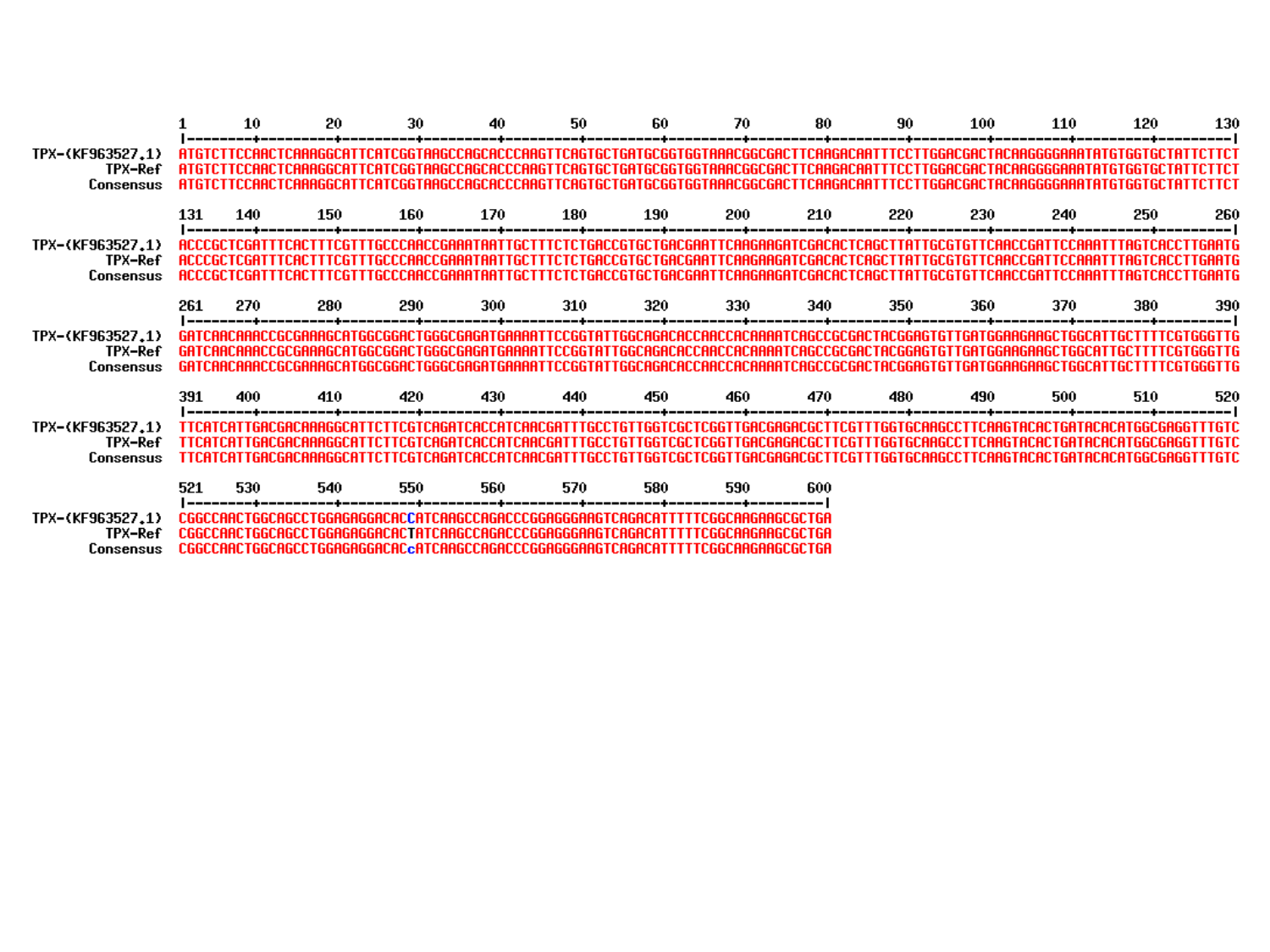

## Slide 16
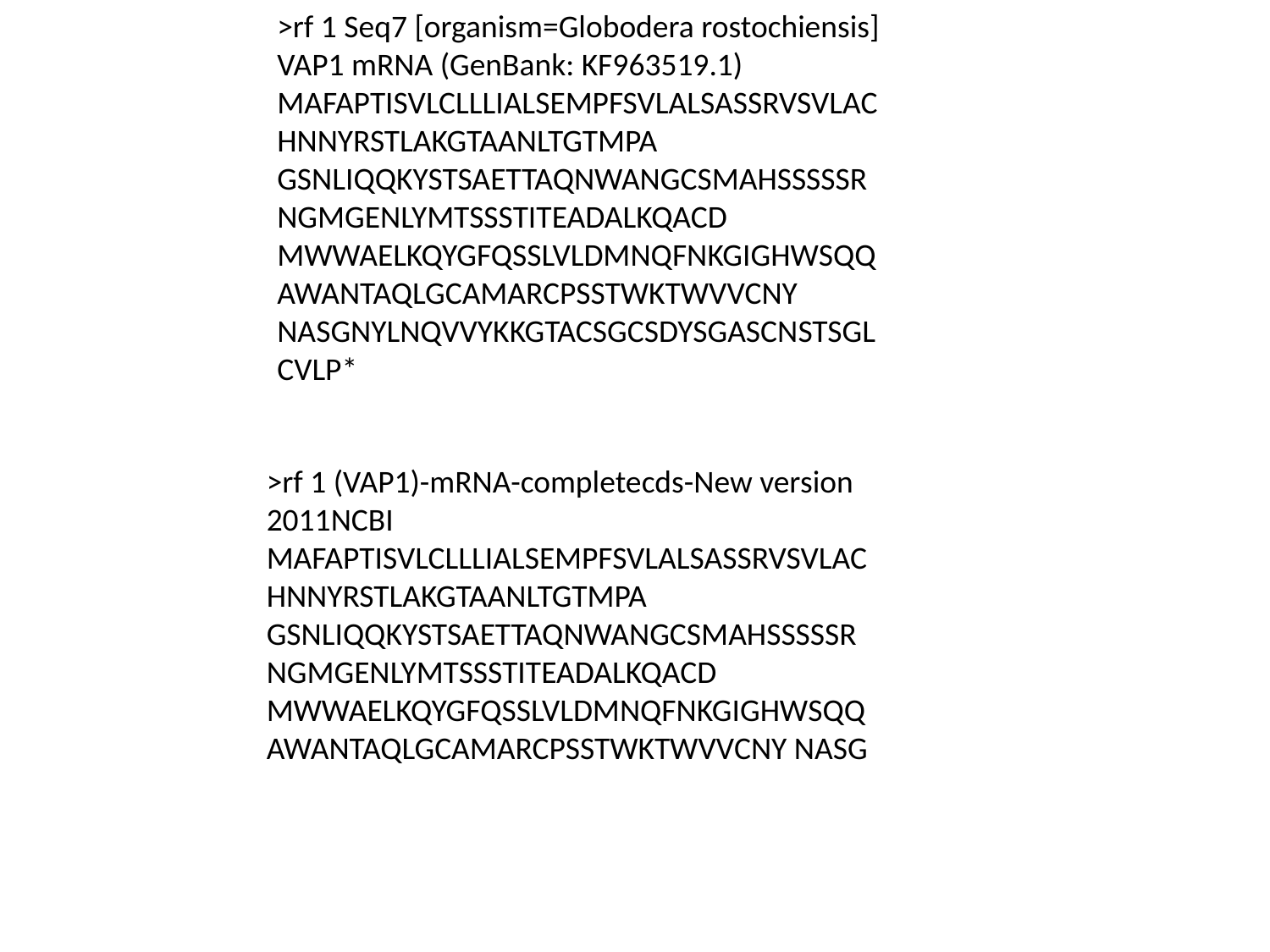

>rf 1 Seq7 [organism=Globodera rostochiensis] VAP1 mRNA (GenBank: KF963519.1) MAFAPTISVLCLLLIALSEMPFSVLALSASSRVSVLACHNNYRSTLAKGTAANLTGTMPA GSNLIQQKYSTSAETTAQNWANGCSMAHSSSSSRNGMGENLYMTSSSTITEADALKQACD MWWAELKQYGFQSSLVLDMNQFNKGIGHWSQQAWANTAQLGCAMARCPSSTWKTWVVCNY NASGNYLNQVVYKKGTACSGCSDYSGASCNSTSGLCVLP*
>rf 1 (VAP1)-mRNA-completecds-New version 2011NCBI MAFAPTISVLCLLLIALSEMPFSVLALSASSRVSVLACHNNYRSTLAKGTAANLTGTMPA GSNLIQQKYSTSAETTAQNWANGCSMAHSSSSSRNGMGENLYMTSSSTITEADALKQACD MWWAELKQYGFQSSLVLDMNQFNKGIGHWSQQAWANTAQLGCAMARCPSSTWKTWVVCNY NASG
