# Supplementary material for: Analysis of Putative Apoplastic Effectors from the Nematode, Globodera rostochiensis, and Identification of an Expansin-Like Protein That Can Induce and Suppress Host Defenses
Source: PLoS One. 2015 Jan 21;10(1):e0115042. doi: 10.1371/journal.pone.0115042 (PMC4301866; doi:10.1371/journal.pone.0115042)
Supplement: S3 Table — (DOCX) [file pone.0115042.s008.docx]

| Primer Name | Sequence (5' > 3') |
| --- | --- |
| attB1 For CLE-4A/4D/1 | GGGGACAAGTTTGTACAAAAAAGCAGGCTTC AGAACCATGGCCAAGAACGCAATGCTTTGC |
| attB1 For CLE-4A/4B/4CΔSP | GGGGACAAGTTTGTACAAAAAAGCAGGCTTC AGAACCATGACGAATAAAAAGGGTGATGAAG  AACC |
| attB2 rev CLE-4A to CLE-4D | GGGGACCACTTTGTACAAGAAAGCTGGGTC TCAGTGATGTATTGGGTCG |
| attB1 for CLE-4B/4C | GGGGACAAGTTTGTACAAAAAAGCAGGCTTC AGAACCATGGCCACGAACACAATGCTTTGC |
| attB1 for CLE-1ΔSP | GGGGACAAGTTTGTACAAAAAAGCAGGCTTC AGAACCATGACAAATGAAAAGGATGATAAAG  AAGC |
| attB2 rev CLE-1 | GGGGACCACTTTGTACAAGAAAGCTGGGTC TCAGCGATGTTGTCGGTCG |
| attB1 for eng-1 | GGGGACAAGTTTGTACAAAAAAGCAGGCTTC AGAACCATGAGTCGACTCCAGTCTCTG |
| attB2 rev eng-1 | GGGGACCACTTTGTACAAGAAAGCTGGGTC TTAGCCCGAACATGCCGTTGTTGAC |
| attB1 for eng-2 | GGGGACAAGTTTGTACAAAAAAGCAGGCTTC AGAACCATGTGTCGTCTCCAGTTCCTG |
| attB2 rev eng-2 | GGGGACCACTTTGTACAAGAAAGCTGGGTC TTAGCCTGATTTGGACTTGG |
| attB2 rev eng-3/4 | GGGGACCACTTTGTACAAGAAAGCTGGGTC TCAACCGCGGCAACTTACTCC |
| attB1 for EXPB1/EXP1 | GGGGACAAGTTTGTACAAAAAAGCAGGCTTC AGAACCATGAGCTCCTCTGAAGCAATTCTGTG |
| attB1 for EXPB1/EXP1ΔSP | GGGGACAAGTTTGTACAAAAAAGCAGGCTTC AGAACCATGCAAATCGTTTTGGCCAGTGTTACG |
| attB2 rev EXPB1 | GGGGACCACTTTGTACAAGAAAGCTGGGTC GCATTTCAAATAGGTGAGC |
| attB2 rev EXP1 | GGGGACCACTTTGTACAAGAAAGCTGGGTC TCAAATAGGTGAGCGTAGC |
| attB1 for EXPB2 | GGGGACAAGTTTGTACAAAAAAGCAGGCTTC AGAACCATGAGCTGCTCCCAATTAATTCTGTG |
| attB1 for EXPB2ΔSP | GGGGACAAGTTTGTACAAAAAAGCAGGCTTC AGAACCATGTGCATGGGCTGTCTGTCGAG |
| attB2 rev EXPB2 | GGGGACCACTTTGTACAAGAAAGCTGGGTC TCAGCAAGCTTTGATGCC |
| attB1 for VAP1/2 | GGGGACAAGTTTGTACAAAAAAGCAGGCTTC AGAACCATGGCATTTGCCCCAACAATTTCTG |
| attB2 rev VAP1 | GGGGACCACTTTGTACAAGAAAGCTGGGTC TTACCCAATGCATGGTAGTTGG |
| attB1 for pel1 | GGGGACAAGTTTGTACAAAAAAGCAGGCTTC AGAACCATGCTTTTTGTTATCATTTCAATAGTTT  TTGCC |
| attB2 rev pel1 | GGGGACCACTTTGTACAAGAAAGCTGGGTC TTAGTTGACAATTTTAATAGCC |
| attB1 for pel2 | GGGGACAAGTTTGTACAAAAAAGCAGGCTTC AGAACCATGCTGCACCATTTATTCG |
| attB2 rev pel2 | GGGGACCACTTTGTACAAGAAAGCTGGGTC TCACTCACAATCACTGATCAGC |
| attB1 for metalP | GGGGACAAGTTTGTACAAAAAAGCAGGCTTC AGAACCATGCATTACGCCAAATTATTTTTTG |
| attB2 rev metalP | GGGGACCACTTTGTACAAGAAAGCTGGGTC TTATTTACCCAAATGGGGCTGTG |
| attB1 for E9 | GGGGACAAGTTTGTACAAAAAAGCAGGCTTC AGAACCATGCATCTTTGCGTTTATTC |
| attB2 rev E9 | GGGGACCACTTTGTACAAGAAAGCTGGGTC TTACTCGCAGTTTATCACC |
| attB1 for ams1 | GGGGACAAGTTTGTACAAAAAAGCAGGCTTC AGAACCATGCAATTTTCTTTCATTATTCTGTC |
| attB1 for ams1ΔSP | GGGGACAAGTTTGTACAAAAAAGCAGGCTTC AGAACCATGTTGCCCCGCCTCAATGAACTG |
| attB2 rev ams1 | GGGGACCACTTTGTACAAGAAAGCTGGGTC TCAGACATCCAACACATCC |
| attB1 for GPX | GGGGACAAGTTTGTACAAAAAAGCAGGCTTC AGAACCATGATTTCATTTTTGCTGATTCC |
| attB2 rev GPX | GGGGACCACTTTGTACAAGAAAGCTGGGTC TTACGATTTTTTCACTTCTCC |
| attB1 for sxp1 | GGGGACAAGTTTGTACAAAAAAGCAGGCTTC AGAACCATGCAAATTCTATTTTCATCAACC |
| attB1 for sxp1ΔSP | GGGGACAAGTTTGTACAAAAAAGCAGGCTTC AGAACCATGCAATTTTCCATACAATGCGTG |
| attB2 rev sxp1 | GGGGACCACTTTGTACAAGAAAGCTGGGTC TTAATTTGGTTTATCCATGC |
| attB1 for A42 | GGGGACAAGTTTGTACAAAAAAGCAGGCTTC AGAACCATGAATGCTTTTATTATTTTTGTC |
| attB2 rev A42 | GGGGACCACTTTGTACAAGAAAGCTGGGTC TCATTTTCGTCTTATGAGCTTGC |
| attB1 for Gr-UBCEP12 | GGGGACAAGTTTGTACAAAAAAGCAGGCTTC AGAACCATGAAGCATTTTGGGCTGATTC |
| attB1 for Gr-UBCEP12ΔSP | GGGGACAAGTTTGTACAAAAAAGCAGGCTTC AGAACCATGCAAATTTTCGTGAAGACACTG |
| attB2 rev Gr- UBCEP12 | GGGGACCACTTTGTACAAGAAAGCTGGGTC TCAGCTTCGGTGGTTCTCG |
| attB1 for Gr-33E05A/B | GGGGACAAGTTTGTACAAAAAAGCAGGCTTC AGAACCATGCGCACCATTCTCTTCTTGG |
| attB1 for Gr-33E05A/BΔSP | GGGGACAAGTTTGTACAAAAAAGCAGGCTTC AGAACCATGGCAACGCCAGAAACATCG |
| attB2 rev Gr-33E05A/B | GGGGACCACTTTGTACAAGAAAGCTGGGTC TCATTCCGTCACAACTGG |
| attB2 rev SKP1 | GGGGACCACTTTGTACAAGAAAGCTGGGTC TCAGTCCTCACACCAGGCATTCTC |
| attR1 for + Cla1 + Xba1 | TCTAGAATCGATACAAGTTTGTACAAAAAAGC |
| attR2 rev + Sal1 | GGATCCGTCGACACCACTTTGTACAAGAAAGC |
| pgR106/pgR107 for | CTAGATGCAGAAACCATAAGG |
| pgR106/pgR107 rev | GAGGTAGTTGACCCTATGG |
| pDONR207 For | TCGCGTTAACGCTAGCATGGATCTC |
| pDONR207 Rev | GTAACATCAGAGATTTTGAGACAC |
| attR1 for + Cla1 + XbaI | TCTAGAATCGATTGAAGCAAGCCTCCTGAGGTACC |
| attR2 rev + xHO1 + AGTAGA | AGTAGACTCGAGGTAGCTTCATCTTGGTACC |
| attB1 for TpXF | GGGGACAAGTTTGTACAAAAAAGCAGGCTTC AGAACCATGTCTTCCAACTCAAAGGCATTC |
| attB2 rev TpXR | GGGGACCACTTTGTACAAGAAAGCTGGGTC TCAGCGCTTCTTGCCGAAAAATGTCTG |
| attB1 for Gr4D06-A2ΔSP | GGGGACAAGTTTGTACAAAAAAGCAGGCTTC AGAACCATGTCTTCTCCCCCTGCAGCACTG |
| attB1 for Gr4D06-A2ΔSP | GGGGACAAGTTTGTACAAAAAAGCAGGCTTC AGAACCATGTGCATTTCGGCCCCACAATTCC |
| attB2 rev Gr4D06-A2 | GGGGACCACTTTGTACAAGAAAGCTGGGTC TCAGAGCTTGTGCGAGCCGGAATCG |
| attB1 for Gr4D06-F10 | GGGGACAAGTTTGTACAAAAAAGCAGGCTTC AGAACCATGGCCAATCTTTCAACTCTGC |
| attB1 for Gr4D06-F10 ΔSP | GGGGACAAGTTTGTACAAAAAAGCAGGCTTC AGAACCATGCTGATCAGCGATTTGTGTCTG |
| attB2 rev Gr4D06-F10 | GGGGACCACTTTGTACAAGAAAGCTGGGTC TTATTGTTTTGTGTAAGCGCTGTG |
| pGBKT7g rev | CAGCTATGACCATGATTACG |
| attB1 for GFP | GGGGACAAGTTTGTACAAAAAAGCAGGCTTC AGAACCatgagtaaaggagaagaacttttcactg |
| attB2 rev GFP | GGGGACCACTTTGTACAAGAAAGCTGGGTC TTATTTGTATAGTTCATCCATGC |
| EXPB2R-HIS-HA -Xba1-Sal1 | ggcgTCTAGAGTCGACCTAGTGGTGATGGTGATGATGAGCGTAATCTGGAACATCGTATGGGTA  TGAGCAAGCTTTGATGCC |
| EXPB2F-Cla1-Not1 | gcatATCGATGCGGCCGCAGAACCATGAGCTGCTCCCAATTAATT |
| EXPB1F-Cla1-Not1 | gcatATCGATGCGGCCGCAGAACC AGAACCATGAGCTCCTCTGAAGCAATTCTGTG |
| EXPB1R-HIS-HA -Xba1-Sal1 | ggcgTCTAGAGTCGACCTAGTGGTGATGGTGATGATGAGCGTAATCTGGAACATCGTATGGGTA  GTATCAAATAGGTGAGCGTAGC |
| GrExpB2_270F | CAGCAGTACATGTTGGACGAC |
| GrExpB2_3UTR_R | ATTGGCATTGTCGAATAAATTG |
| GrEng1_1157F | TGTCTCAACCAACACATGGAAC |
| GrEng1_1337R | TTTATGTAGCTGGGCAGTGTGT |
| GrPel1_571F | ATTTCGGCTAACTCGAACTACG |
| GrPel1_770R | ATAGCCGAAGTGCTGTAGGAAC |
| GrSkp1_209F | ACATCACAAGGATGATGCTC |
| GrSkp1_465R | CTCGGGAGTAAAGTCGTTTTTG |
| GrActin-c660Fb | CGACTTCGAGCAGGAAAT |
| GrActin-R2b | ATGTCGATGTCGCACTTC |

**Supplemental Table 3.** Primer sets used for cloning and qRT-PCR of *G. rostochiensis* effectors
